# Comprehensive expression analysis with cell-type-specific transcriptome in ALS-linked mutant SOD1 mice: Revisiting the active role of glial cells in disease

**DOI:** 10.3389/fncel.2022.1045647

**Published:** 2023-01-04

**Authors:** Hirofumi Yamashita, Okiru Komine, Noriko Fujimori-Tonou, Koji Yamanaka

**Affiliations:** ^1^Department of Neurology, Japanese Red Cross Wakayama Medical Center, Wakayama, Japan; ^2^Department of Neurology, Graduate School of Medicine, Kyoto University, Kyoto, Japan; ^3^Department of Neuroscience and Pathobiology, Research Institute of Environmental Medicine, Nagoya University, Nagoya, Japan; ^4^Support Unit for Bio-Material Analysis, RRD, RIKEN Center for Brain Science, Wako, Japan; ^5^Department of Neuroscience and Pathobiology, Nagoya University Graduate School of Medicine, Nagoya University, Nagoya, Japan; ^6^Institute for Glyco-Core Research (iGCORE), Nagoya University, Nagoya, Japan

**Keywords:** transcriptome, amyotrophic lateral sclerosis, microarray, neurodegeneration, astrocytes, microglia, superoxide dismutase 1 (SOD1), lipids/lipoproteins

## Abstract

Non-cell autonomous mechanisms are involved in the pathogenesis of amyotrophic lateral sclerosis (ALS), an adult neurodegenerative disease characterized by selective motor neuron loss. While the emerging role of glial cells in ALS has been noted, the detailed cell-type-specific role of glial cells has not been clarified. Here, we examined mRNA expression changes using microarrays of the spinal cords of three distinct lines of mutant superoxide dismutase (SOD) 1 transgenic mice, an established ALS model. Our analysis used a transcriptome database of component cell types in the central nervous system (CNS), as well as SOD1^*G*93*A*^ cell-type transcriptomes. More than half of the differentially expressed genes (DEGs) were highly expressed in microglia, and enrichment analysis of DEGs revealed that immunological reactions were profoundly involved and some transcription factors were upregulated. Our analysis focused on DEGs that are highly expressed in each cell type, as well as chemokines, caspases, and heat shock proteins. Disease-associated microglial genes were upregulated, while homeostatic microglial genes were not, and galectin-3 (Mac2), a known activated microglial marker, was predicted to be ectopically expressed in astrocytes in mutant SOD1 mice. In mutant SOD1 mice, we developed a prediction model for the pathophysiology of different cell types related to TREM2, apolipoprotein E, and lipoproteins. Our analysis offers a viable resource to understand not only the molecular pathologies of each CNS constituent cell type, but also the cellular crosstalk between different cell types under both physiological and pathological conditions in model mice for various neurodegenerative diseases.

## 1. Introduction

Amyotrophic lateral sclerosis (ALS) is an adult neurodegenerative disease characterized by the selective loss of motor neurons. Since non-cell autonomous mechanisms have been shown to be involved in the pathology of ALS (Boillée et al., 2006; Yamanaka et al., 2008), it is important to analyze the glial cells surrounding motor neurons such as microglia and astrocytes, in addition to the degenerating motor neurons, to elucidate the pathophysiology.

Mice overexpressing the ALS-linked mutant superoxide dismutase (SOD) 1 gene [mutant SOD1 mice (Gurney et al., 1994; Wong et al., 1995; Bruijn et al., 1997)] are a well-characterized rodent model for ALS, allowing a time-course pathological analysis of the spinal cord where motor neuron loss and subsequent neuroinflammation are observed. Since the spinal cord is composed of various cell types, such as motor neurons, non-motor neurons, astrocytes, microglia, oligodendrocytes, and vascular endothelial cells, mRNA expression analysis using the whole spinal cord involves two major problems: it is not clear which cell types are responsible for the alteration in mRNA levels, and the ratio of constitutive cells may vary between experimental and control groups. In the spinal cords of mutant SOD1 mouse, activation of microglia and astrocytes as well as a decrease in the number of motor neurons is observed (Beers and Appel, 2019); therefore, the two major problems mentioned above must be considered. One solution to these problems is to isolate the particular cell types and perform an expression analysis by each cell type, and indeed, remarkable results have been obtained by this method (Sun et al., 2015). However, sometimes there are problems associated with cell separation such as the deterioration of mRNA quality during the isolation procedure, and, especially in human postmortem samples, the inability to manipulate genes for isolation using tags. Another solution is population-specific expression analysis (PSEA), a sophisticated method that processes and analyzes the tissue as a whole (Kuhn et al., 2011), but PSEA has some restrictions in that the genes for analysis must be highly expressed in specific cell types, and the expression levels of selected references require at least moderate variation among samples.

Therefore, we chose a new method for first measuring mRNA expression changes in spinal cord samples composed of various cell types, then referring to an independently prepared transcriptome database for each constituent cell type to determine the relative expression levels among component cell types. In addition, we also obtained and analyzed data from SOD1^*G*93*A*^ cell-type transcriptomes. We report here that this method allowed for greatly improved analysis of spinal cord samples in mutant SOD1 mice to investigate ALS pathology.

## 2. Materials and methods

### 2.1. Transgenic mice

Transgenic mice expressing inherited ALS-linked human ^*SOD1G*85*R*^ and *SOD1^WT^* genes (B6.Cg-Tg(SOD1*G85R)148Dwc/J and B6.Cg-Tg(SOD1)76Dwc/J) were kind gifts from Dr. Don Cleveland (University of California, San Diego, CA, United States) (Wong et al., 1995). LoxSOD1^*G*37*R*^ mice (B6.Cg-Tg(SOD1*G37R)1Dwc/J) were previously described (Boillée et al., 2006). Briefly, loxSOD1^*G*37*R*^ mice express mutant SOD1^*G*37*R*^ protein ubiquitously, and their phenotype is essentially indistinguishable from previously described SOD1^*G*37*R*^ lines in the absence of Cre recombinase (Wong et al., 1995). We did not express Cre recombinase in any tissues or cells in these experiments. All SOD1 transgenic mice express the human *SOD1* gene including its endogenous promotor; therefore, human SOD1 protein is expressed ubiquitously. Genotyping of human SOD1 transgenic mice was described elsewhere (Komine et al., 2018). Mice were maintained under a standard specific pathogen-free environment (12-h light-dark cycle) with free access to food and water, and were treated in compliance with the guidelines established by the Institutional Animal Care and Use Committee of Nagoya University and RIKEN. The study using genetically modified animals was approved by the Animal Care and Use Committee and Recombinant DNA Experiment Committee of Nagoya University and RIKEN.

### 2.2. Microarray analysis of mutant SOD1 transgenic mice

Total RNA was isolated from lumbar spinal cords, where the motor neurons are remarkably degenerated, of 11-month-old loxSOD1^*G*37*R*^ and 11-month-old SOD1^*G*85*R*^ mice (at symptomatic stage) as well as age-matched SOD1*^WT^* mice and C57BL/6 littermates as controls using TRIzol (Life Technologies, Carlsbad, CA, United States) followed by an RNeasy Mini Kit (Qiagen, Valencia, CA, United States). We used spinal cord samples at the symptomatic stage, because the onset time and progression rate in ALS-linked mutant SOD1 mice are determined by the damage within motor neurons and glial cells (microglia and astrocytes), respectively (Boillée et al., 2006; Yamanaka et al., 2008). The quality of extracted RNA was verified by its purity (i.e., A_260/290_ ratio > 1.9) and integrity (RNA integrity number (RIN) > 7.3, measured with an Agilent 2100 bioanalyzer). Two micrograms of total RNA was first reverse transcribed into cDNA, then the cDNA was converted to biotinylated cRNA. Fifteen micrograms of biotinylated cRNA was hybridized with a Mouse Genome 430_2.0 Array (GeneChip^®^, Affymetrix, Santa Clara, CA, United States) according to the standard protocol available from Affymetrix.

For analysis of the GeneChip data, CEL files obtained using GCOS v1.2 (Affymetrix) were analyzed with GeneSpring GX v14.9 software (Agilent Technologies, Santa Clara, CA, United States), whereas “Present/Marginal/Absent” calls were calculated with GCOS. In GeneSpring GX, all CEL files were normalized with the MAS5 algorithm. Comparison analysis between three mutant and three control samples was performed with GeneSpring GX using entities that were called “Present” or “Marginal” on at least three out of all six samples in the mutant group and control group combined (FLAG-call criteria). An entity is a discrete probe set onto a gene. For comparison of SOD1^G93A^ and littermate controls (postnatal day 112, symptomatic stage) (Lerman et al., 2012), we downloaded GeneChip raw data (CEL files) deposited at the Gene Expression Omnibus (GEO) Database^1^ and processed them in the same way as our CEL files derived from SOD1^G37R^, SOD1^G85R^, and SOD1^WT^, and non-transgenic mice. For all gene names, NetAffx Annotation Update 32 was used (Affymetrix). Enrichment analysis of a gene list was performed using the PANTHER (Protein ANalysis THrough Evolutionary Relationships) classification system^2^ for analysis of large-scale genome-wide experimental data.

### 2.3. Generating an integrated cell-type-specific transcriptome database

Cell type-specific transcriptomes were prepared by obtaining CEL files in which specific cell types (i.e., motor neurons, neurons, astrocytes, microglia, and oligodendrocytes of mice) were independently analyzed with a microarray (Cahoy et al., 2008; Noristani et al., 2015). All CEL files of five cell types, which were generated using the same Mouse Genome 430_2.0 Array, were then normalized by the MAS5 algorithm (Hubbell et al., 2002), which is a global normalization method that assumes the overall amount of mRNA measured from each sample is constant, for comparing relative expression levels of mRNAs of all genes among the five major cell types in the CNS.

### 2.4. Analysis of disease-specific microglial/astrocyte/motor neuronal microarray data

GeneChip raw data (CEL files) of disease-specific (SOD1^G93A^ and control littermates) isolated microglia (Noristani et al., 2015), astrocytes (Baker et al., 2015), and motor neurons (Nardo et al., 2013; Trolese et al., 2020) were obtained from the GEO Database and were normalized by the MAS5 algorithm and calculated for fold changes.

### 2.5. Statistical analysis

**FIGURE 1:**
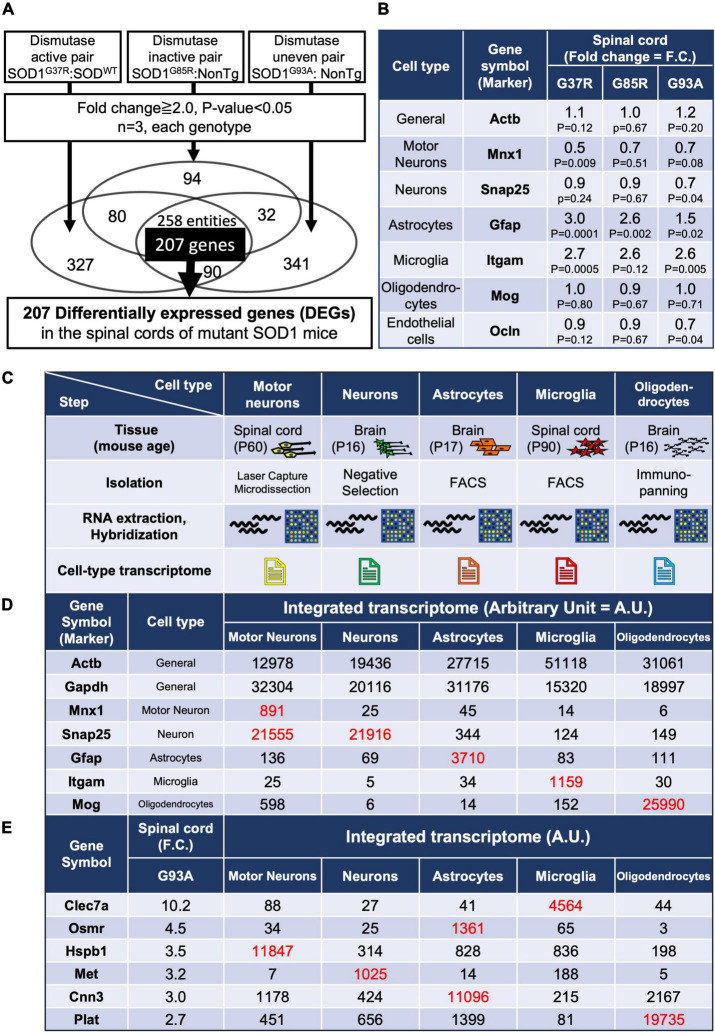
Gene expression analysis of whole spinal cords in mutant SOD1 mice, and establishment of an integrated transcriptome. **(A)** Experimental design to identify genes in the lumbar spinal cords of mutant SOD1 mice that are reliably changed by mutant SOD1 toxicity, independent of SOD1 activity. A total of 207 overlapping, differentially expressed genes (DEGs) were extracted that were commonly changed by comparison in three pairs. **(B)** Gene expression changes in general or in cell-type marker genes. **(C)** Characteristics of each cell type sample used to create the integrated transcriptome. Motor neurons were obtained by Laser Capture Microdissection from spinal cord. Neurons were obtained by negative selection using residual samples after removal of oligodendrocyte lineage cells and microglia by immunopanning, astrocytes by FACS, and endothelial cells and pericytes by immunopanning, from brain cell suspensions. Reported cell purity, negative for an astrocyte marker (S100β), oligodendrocyte markers (GalC, MOG, and O1), and a microglial and pericytic marker (BSL1 lectin), was >99.9%. Astrocytes were obtained from S100β-EGFP positive cells purified by FACS after the removal of oligodendrocytes by immunopanning from brain cell suspensions. Reported cell purity was >99.5% by S100β-EGFP positivity. Microglia were obtained from CD11b-positive cells purified by FACS from dissociated cells from lumber spinal cords. Oligodendrocytes were obtained by anti-MOG immunopanning from brain cell suspensions, which yielded 100% MOG-positive, >95% MBP-positive, and 0% NG2-positive cells. **(D)** The data from the CEL file of each cell type were normalized to show the relative expressions of each gene in each cell type (integrated transcriptome). As shown in red (hereafter, red font shows values to be focused on along with our explanation), the marker genes of each cell type itself were highly enriched. **(E)** The integrated transcriptome of representative genes in 207 DEGs shows that *Clec7a, Osmr* and *Cnn3, Hspb1, Met*, and *Plat* are expressed at relatively high levels in microglia, astrocytes, motor neurons, neurons, and oligodendrocytes, respectively. FACS, fluorescence-activated cell sorting.

**FIGURE 2:**
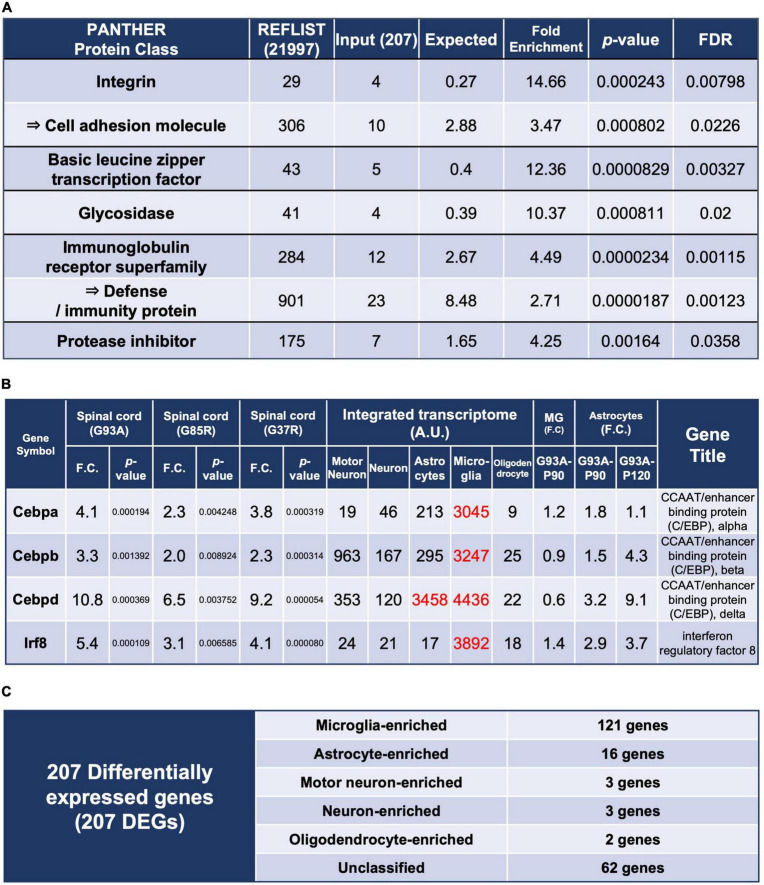
Enrichment analysis using the 207 DEGs and classification of each of the 207 DEGs by cell types with high expression. **(A)** Enrichment analysis using the 207 DEGs as an input. The first column shows the name of the PANTHER classification category. The second column shows the number of genes in the reference list that map to this particular PANTHER classification category. The total number of genes in the reference list is 21,997. The third column shows the observed number of genes in our input list that map to this PANTHER classification category. The fourth column shows the expected value, which is the number of genes we would expect in our list for this PANTHER category, based on the reference list. The fifth column shows the fold enrichment, which is the ratio of the value of column 3 (input: observed number) over that of column 4 (expected number). The sixth column shows the raw *p*-values. The seventh column shows the *q*-value (adjusted *p*-value, reflecting the false discovery rate) as calculated by the Benjamini-Hochberg procedure. *Cell adhesion molecule* is in the parent category of integrin, so it is shown in the row below. Similarly, *defense/immunity protein* is the parent category of *immunoglobulin receptor superfamily*. Therefore, they are indicated with arrows. **(B)** Representative transcription factors in the 207 DEGs. Three CCAAT/enhancer binding proteins and interferon regulatory factor 8 are shown. **(C)** All of the 207 DEGs were classified into cell types in which each gene is highly expressed; *Unclassified* are the genes that are not highly expressed in one particular cell type. MG, microglia.

**FIGURE 3:**
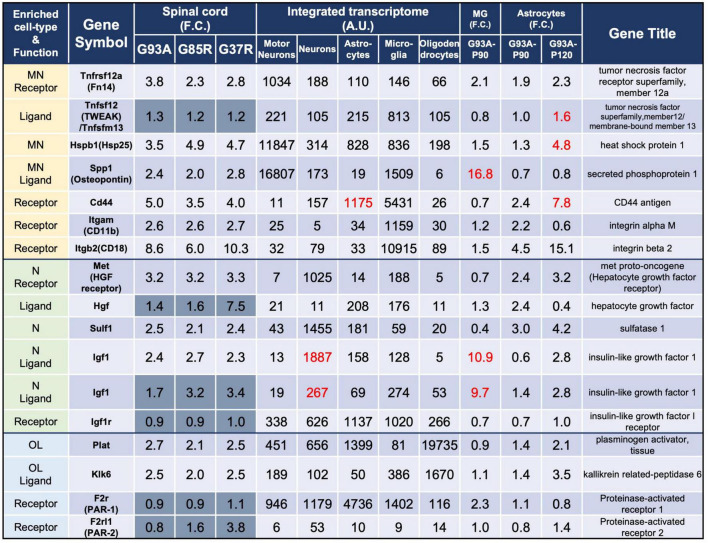
Representative motor neuron-enriched genes, neuron-enriched genes, and oligodendrocyte-enriched genes in 207 DEGs were analyzed using the integrated transcriptome and SOD1^G93A^ cell-type transcriptome. The 3rd to 5th columns show the fold changes in each gene expression in SOD1^G93A^, SOD1^G85R^, and SOD1^G37R^ mouse spinal cords compared to control samples, respectively. Genes with dark gray backgrounds in the 3rd to 5th columns that indicate fold changes (F.C.) are not included in the 207 DEGs. The 6th to 10th columns show the integrated transcriptome. The 11th column shows the fold change in expression of each gene in P90 SOD1^G93A^ microglia relative to control microglia. The 12th to 13th columns show the fold change in expression of each gene in SOD1^G93A^ astrocytes (P90 or P120) relative to control astrocytes. MN, motor neurons; N, neurons; MG, microglia; OL, oligodendrocytes.

**FIGURE 4:**
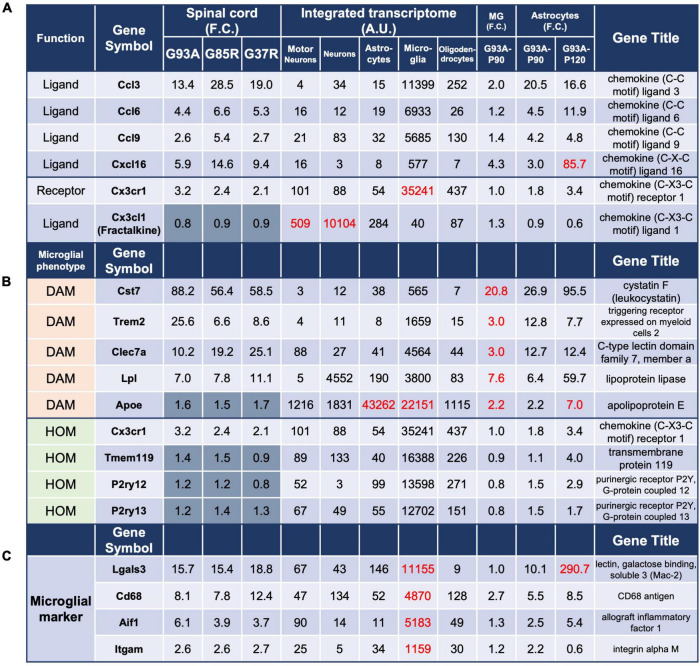
Analysis of genes in 207 DEGs using integrated and SOD1^G93A^ cell-type transcriptomes. The gene expressions for panel **(A)** chemokines and their receptors, **(B)** microglial phenotype: DAM and HOM, and **(C)** ectopic expression of *Lgals3* are shown. The description of each column is the same as in Figure 3. DAM, disease-associated microglia; HOM, homeostatic microglia; MG, microglia.

**FIGURE 5:**
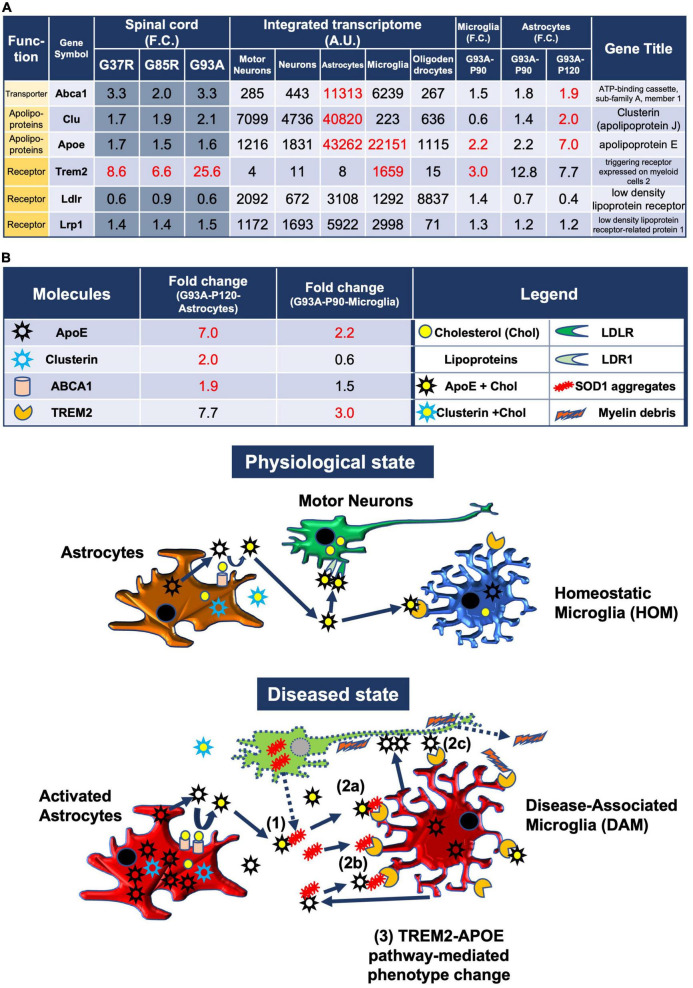
Model of the pathomechanism among different cell types in spinal cords of mutant SOD1 mice related to TREM2, apolipoprotein E, and lipoproteins. **(A)** Gene expression analysis of the relevant transporter *Abca1*, apolipoproteins *Apoe* and *Clu*, and receptors *Trem2, Ldlr*, and *Lrp1*, using integrated and SOD1^G93A^ cell type transcriptomes. **(B)** Physiological state: cholesterol is transported from astrocytes to motor neurons and microglia *via* lipoproteins. Diseased state: (1) Mutant SOD1 aggregates are released from diseased motor neurons and bind to lipoproteins in the intercellular space. (2a) Microglia phagocytose SOD1 aggregate-lipoprotein complexes *via* TREM2. (2b) Microglia phagocytose SOD1 aggregates with or without ApoE *via* TREM2. (2c) Microglia phagocytose ApoE-bound motor neurons *via* TREM2. (3) TREM2-mediated phagocytosis changes the phenotype from HOM to DAM in microglia *via* ApoE signaling. Excessive DAM activation may contribute to exacerbation of ALS pathology.

Statistical analysis for 207 differentially expressed genes (DEGs) was performed using an unpaired *t*-test with Benjamini and Hochberg’s multiple testing correction at a false discovery rate of 0.05. Fold changes as shown in Figure 1B and Figure 2B are listed with *p*-values, and in Figures 3–5, *p*-values for the 207 DEGs are not shown. Instead, *p*-values for all 207 DEGs are listed in Supplementary Table 5.

## 3. Results and discussion

### 3.1. mRNA expression analysis of the spinal cords of three SOD1-ALS mouse models

In general, microarray experiments often detect a very large number of differentially expressed genes (DEGs), making it difficult to extract the genes involved in some pathological conditions. In addition, most of the SOD1-ALS mouse microarray data available in public database are the ones from high copy SOD1^G93*A*^-overexpressing mice, in which SOD1 activity is very high. Some DEGs may be resulted from high SOD1 activity, when a wild-type mouse is used as the control. Therefore, we designed three sets of comparisons considering the SOD1 activity as described below.

There are several mouse models that overexpress the human *SOD1* gene carrying pathogenic mutations at different amino acid sites, such as G93A, G37R, and G85R mutations, which are also found in human inherited ALS. While the disease courses of these three types of mutant SOD1-overexpressing mice differ due to differences in SOD1 expression levels, they all share the common feature of progressive paralysis of limbs, which is a hallmark of ALS. SOD1^G93A^ and SOD1^G37R^ retain Cu/Zn superoxide dismutase (SOD1) activity, while SOD1^G85R^ is inactive (Borchelt et al., 1994; Yim et al., 1996). Since pathogenicity in SOD1-ALS is mediated through a gain of toxicity from mutant SOD1 proteins—unrelated to the loss or gain of dismutase enzymatic activity—(Dal Canto and Gurney, 1995; Reaume et al., 1996), we profiled the mRNA expression of lumbar spinal cords from three lines of SOD1-ALS mice. Comparisons were conducted in three ways: paired excessive dismutase activity, normal dismutase activity, and uneven dismutase activity (Supplementary Table 1). Dismutase activity of comparison 2 was strictly the same due to having only endogenous mouse SOD1 (C57BL/6) and the transgene without SOD1 activity (SOD1^G85R^), while the increased dismutase activity of comparison 1 was not strictly adjusted. Comparison 3 used public deposited data. We identified 258 entities (probe sets) with more than twofold changes in the three different comparisons in common. Since the 258 entities included some duplications from the same gene (due to multiple probe sets per transcript), we sorted out and extracted 207 DEGs without duplications (Figure 1A).

### 3.2. The shift in the expression of cell type markers indicates a decreased number of neurons and increased activation of astrocytes and microglia

Prior to the analysis of DEGs, the expression levels of markers of the major cell types that constitute the spinal cord were examined. We found a significant decrease or a decreasing trend in the motor neuronal marker *Mnx1* (motor neuron and pancreas homeobox 1) depending on each mutant SOD1 mouse, indicating a loss of motor neurons, and significant upregulation or upregulation trend of *Gfap* and *Itgam*, indicating activation or proliferation of astrocytes and microglia (Figure 1B and Supplementary Figure 1). These results are consistent with previous reports (Beers and Appel, 2019). In contrast, a marker for oligodendrocytes showed no change (Figure 1B). This might be because there are lots of oligodendrocytes in spinal cords other than ones in contact with motor neurons, so therefore marker analysis of spinal cord samples might not reflect disturbances of oligodendrocytes, despite that degeneration of oligodendrocytes has been reported in the ventral spinal cords of SOD1*^G93A^* mice (Kang et al., 2013).

### 3.3. Establishment of an integrated cell-type-specific transcriptome database constituting the central nervous system, with additional SOD1^G93A^ cell-type transcriptomes

Since the microarray used in our experiment is considered one of the definitive final versions for analyzing mouse mRNA expression, there is plenty of data (i.e., over 58,000 samples and 4,400 experiments) using microarrays of the same type (i.e., Affymetrix Mouse Genome 430 2.0 Array) in public databases, which allowed us to directly compare expression data among different experiments.

The distribution of genes (*y*-axis) across the gene expression level (*x*-axis) was different among different cell types (Supplementary Figure 2A; MAS5 normalization) as has been previously reported (Holtman et al., 2015), and it is difficult to determine which normalization method is optimal. This is because each normalization method has its own assumptions. For example, MAS5, which is a representative global normalization, assumes that the total amount of mRNA measured from each sample is constant, while robust multiarray average (RMA), which is a representative quantile normalization, assumes that the signal intensity is the same for the same expression rank. It is not clear which assumption is preferable in downstream analyses between different cell types. Normalization methods, including MAS5 and RMA, have their own advantages and disadvantages (Choe et al., 2005; Lovén et al., 2012; Liu et al., 2019). We chose MAS5 mainly for two reasons: (1) the fold changes between the two experimental groups are less likely to be underestimations than the actual values (Pepper et al., 2007), and (2) expression values in samples already analyzed are invariant even if samples (for example lymphocytes) are added for new analyses in the future, since MAS5 normalizes on a per-array basis, while RMA does so on a per multi-array basis.

An integrated cell-type-specific transcriptome database was created using publicly available data (Supplementary Table 2A) derived from the same version of Affymetrix arrays for motor neurons, neurons, astrocytes, microglia, and oligodendrocytes (Figure 1C). Open access available data were limited, so mouse age and isolation method were different among each cell type. However, it still remains practical to compare transcriptomes between different cell types. That is because Cahoy et al. have reported that a dendrogram reflecting transcriptomes among different CNS cell type (astrocytes, neurons, and oligodendrocytes) shows strong similarity within one cell type despite different mouse ages, and large differences between astrocytes, neurons, and oligodendrocytes (Cahoy et al., 2008).

Comparisons of the expression values of specific markers for each cell type validated the specificity of each transcriptome used for generating the integrated cell-type-specific transcriptome (hereafter named an integrated transcriptome) (Figure 1D). For each DEG that was identified in the mutant SOD1 mouse spinal cord, the relative expression levels of each DEG in the constituent cells can be determined by referring to the integrated transcriptome (Figure 1E).

To validate the cell-type specificity of our integrated transcriptome database, we confirmed the expressions of several genes among the 207 DEGs by immunohistochemistry (Supplementary Figure 1). Also, it was tested for previously reported microglia-specific marker genes (Chiu et al., 2013). Although the degree of enrichment varied, we confirmed a general tendency for expression to be highly enriched in microglia (Supplementary Table 3). The discrepancies in the degree of microglial enrichment may reflect differences both in the samples for CNS cell types and in the methods including measurement (microarray vs. RNA-seq) and normalization (MAS5 vs. RPKM). Chiu et al. (2013) pointed out that *Olfml3, Tmem119*, and *Siglech* are microglia-specific markers that are highly expressed in CNS microglia, but are rarely expressed in macrophages or monocytes. Our transcriptome database confirmed that these three genes are highly enriched in CNS microglia (Supplementary Table 3).

We also obtained and referenced transcriptome data with cell-type-specific samples isolated from the spinal cords of SOD1^G93A^ mice. Publicly available transcriptome data from Gene Expression Omnibus were obtained for microglia (P90 early symptomatic SOD1^G93A^ mice and controls), astrocytes (P90 early symptomatic and P120 late-symptomatic SOD1^G93A^ mice and controls), and, as shown in the Supplementary Tables, motor neurons (P56 pre-symptomatic, P101 early symptomatic, P111 symptomatic, and P121 late-symptomatic SOD1^G93A^ mice and controls) (Supplementary Tables 2B–D). In the tables (Figures 2B, 3–5 and Supplementary Tables 4, 5, 7–9), we show transcriptomic fold changes of SOD1^G93A^ cells compared to the controls. When interpreting the fold changes of the SOD1^G93A^ cell-type transcriptome, it should be noted that astrocytes (Baker et al., 2015) and motor neurons (Nardo et al., 2013) were isolated by laser capture microdissection (LCM), and therefore, they are somewhat contaminated by other adjacent cell types (Supplementary Table 4).

### 3.4. Enrichment analysis of 207 DEGs suggests immunological reactions and activation of some transcription factors in spinal cords

We performed enrichment analysis with respect to molecular function classification (Mi et al., 2021; Figure 2A), and a total list of the 207 DEGs as classified by the PANTHER system is presented in Supplementary Table 5.

The enrichment of the *integrin and immunoglobulin receptor superfamily* suggested involvement in immunological reactions in spinal cords. Enrichment of *basic leucine zipper transcription factor* was mainly caused by upregulation of CCAAT-enhancer-binding proteins (C/EBPs) (Figure 2B), which regulate innate immunity and proinflammatory genes. C/EBPβ and C/EBPδ have been reported to be elevated in the microglia of SOD1^G93A^ mice (Valente et al., 2012) and in spinal cords of ALS patients (Valente et al., 2013), respectively.

There are five transcription factors other than C/EBPs among the 207 DEGs, of which interferon regulatory factor 8 (*Irf8*) is most notable. The integrated transcriptome shows that Irf8 is expressed almost exclusively in microglia in the CNS (Figure 2B). Irf8 is essential for microglial activation in neurodegeneration (Zhou et al., 2019), and Irf8 is upregulated and contributes to disease progression in a mouse model of experimental autoimmune encephalomyelitis (Yoshida et al., 2014). Therefore, proper control of Irf8 may be a target to ameliorate disease in mutant SOD1 mice.

### 3.5. Most of the 207 DEGs in SOD1-ALS mouse spinal cords are expressed predominantly in microglia, followed by astrocytes

We included *Actb* and *Gapdh*, ubiquitously expressed genes, as ubiquitous markers in the integrated transcriptome database. An approximately fourfold difference in expression of *Actb* was observed between microglia and motor neurons (Figure 1D). It is possible that the actual expression levels differ by about fourfold, but the normalization may have resulted in a nearly fourfold variation. In fact, single-cell RNA-seq of human CNS cells reveals an approximately threefold difference in *ACTB* or *GAPDH* expression levels between high- and low-expressing cell types, respectively (Karlsson et al., 2021; Supplementary Table 6). Therefore, the results of the present analysis should be interpreted with this level of error tolerance. Furthermore, in the Human Protein Atlas^3^ (Sjöstedt et al., 2020), which is an open access resource for human proteins, “cell type enriched genes” are defined as having at least a fourfold higher expression level in one cell type as compared with any other analyzed cell type. We adopted the same criterion, and more than half of the 207 DEGs were classified as enriched in microglia, followed by astrocytes. A small number of enriched genes were also observed in motor neurons, neurons, and oligodendrocytes (Figure 2C).

### 3.6. Analysis of motor neuron-, neuron-, or oligodendrocyte-enriched DEGs

We analyzed all eight DEGs enriched in motor neurons (*Tnfrsf12a, Hspb1*, and *Spp1*), neurons (*Met, Sulf1*, and *Igf1*), or oligodendrocytes (*Plat* and *Klk6*). In this section, we first point out the cell-type-enriched genes that were differentially expressed in our analysis at the beginning of the subsections. The fold changes of the genes in the spinal cords of three mutant SOD1 lines, the integrated transcriptome of the genes, and the fold changes of the genes in the SOD1^G93A^ microglia and astrocytes are displayed in the Figures. We then discuss the gene that we focus on according to previous reports. In the tables of Figures 3–5, in order to distinguish the 207 DEGs from other genes, the background of cells displaying fold changes (F.C.) of other genes in mutant SOD1 mouse spinal cord is dark gray for visual clarity.

All eight DEGs enriched in motor neurons, neurons, or oligodendrocytes have been previously reported to be associated with neurodegenerative diseases including ALS, and genetic manipulation experiments using SOD1^G93A^ mice have been reported for five genes individually or in related signaling pathways as described below.

#### 3.6.1. Motor neuron-enriched DEGs in SOD-ALS mice

The three genes that are enriched in motor neurons and included in the 207 DEGs were *Tnfrsf12a, Hspb1*, and *Spp1* (Figure 3).

*Tnfrsf12a*-encoded fibroblast growth factor-inducible 14 (Fn14) is a TWEAK (tumor necrosis factor (TNF)-like weak inducer of apoptosis) receptor, and the TWEAK-Fn14 pathway functions as a cell death or a neuronal cell death pathway (Nakayama et al., 2003; Potrovita et al., 2004). However, Fn14 has effects on neurite outgrowth without stimulation of its TWEAK ligand (Tanabe et al., 2003). Expression data for TWEAK, the ligand for Fn14, is also shown in the list, but there was no more than a 1.5-fold increase in diseased SOD1-ALS mouse spinal cord. This pathway has already been examined in SOD1^G93A^ mice, and TWEAK has been shown to be elevated in astrocytes of SOD1^G93A^ mice. Our analysis with P120 SOD1^G93A^ astrocytes also shows a 1.6-fold increase in *Tnfsf12*, which encodes TWEAK. Genetic ablation of TWEAK in SOD1^G93A^ mice significantly reduces astrogliosis and microgliosis, and ameliorates skeletal muscle atrophy, but does not extend lifespan and motor neuron survival (Bowerman et al., 2015).

*Hspb1*-encoded heat shock protein 25 (Hsp25) is a small heat shock protein functioning as a chaperone. Immunohistochemistry has shown that Hsp25 immunoreactivity is present in all cranial nerve motor nuclei and the spinal motor neurons in wild-type mice (Maatkamp et al., 2004), as is reflected by our integrated transcriptome. The level of Hsp25 is post-transcriptionally reduced in SOD1^G93A^ mice even before motor neuron loss, and its expression is upregulated in glia at the symptomatic stages (Vleminckx et al., 2002; Maatkamp et al., 2004), which is consistent with our results in which P120 SOD1^G93A^ astrocytes showed a 4.8-fold increase in *Hspb1.* SOD1^G93A^ mice overexpressing Hsp27, which is encoded by *HSPB1* and is a human ortholog of Hsp25, show no effect on motor neuron loss or lifespan (Krishnan et al., 2008).

*Spp1*-encoded osteopontin is a multifunctional protein that is detected in motor neurons with relatively large somas, but not in those with smaller somas (Misawa et al., 2012). Intriguingly, osteopontin is expressed in the motor neuron subtypes fast-twitch fatigue-resistant (FR) and slow-twitch fatigue-resistant (S), which are less vulnerable than fast-twitch fatigable (FF) motor neurons in ALS (Morisaki et al., 2016). Furthermore, osteopontin is also expressed in microglia (Rosmus et al., 2022), which was confirmed by our integrated transcriptome. Osteopontin functions as a ligand, and its main receptors are CD44 and integrin receptors (mainly αMβ2 integrin, also known as CD11b/CD18 integrin in microglia), which are shown in the list for reference. CD44, CD11b, and CD18 were all included in the 207 DEGs and are classified as microglia-enriched genes. CD44, a receptor for osteopontin, is expressed in astrocytes as well as in microglia and has been reported to be involved in astrocytic migration to degenerative motor neuron sites (Morisaki et al., 2016). It has been reported that the elevation of osteopontin is observed primarily in spinal cord microglia of early symptomatic mutant SOD1 mice (Chiu et al., 2013) as revealed by the 16.8-fold change in P90 SOD1^G93A^ microglia. Osteopontin has been shown to have an opsonin effect when microglia exhibit phagocytosis in mutant SOD1 mice and deletion of osteopontin in SOD1^G93A^ mice has been shown to have no effect on survival, but a delayed trend in muscle weakness (Morisaki et al., 2016).

#### 3.6.2. Neuron-enriched DEGs in SOD-ALS mice

The three genes that are enriched in neurons and included in the 207 DEGs were *Met, Sulf1*, and *Igf1* (Figure 3).

*Met*-encoded hepatocyte growth factor (HGF) receptor works as a receptor for HGF. HGF receptor is immunopositive in motor neurons of the facial nerve nucleus (Kadoyama et al., 2009) and anterior horn of spinal cords (Sun et al., 2002) in mice, though *Met* mRNA was hardly expressed in motor neurons in the list. Also, in the human spinal cord, immunoreactivity in anterior horn motor neurons has been reported for both HGF and HGF receptor (Kato et al., 2003), each of which has been shown to prolong lifespan when overexpressed in SOD1^G93A^ mice (Sun et al., 2002; Genestine et al., 2011).

*Sulf1*-encoded extracellular sulfatase (Sulf) 1 regulates the expression of highly sulfated domains of heparan sulfate (HS), also known as HS S-domains, at the cell surface. HS S-domains are accumulated in amyloid plaques in the brains of Alzheimer’s disease (AD) patients, although whether HS S-domains are a common co-depositing factor in protein aggregation diseases remains unknown (Nishitsuji, 2018).

*Igf1*-encoded insulin-like growth factor (Igf) 1 is a neurotrophic and anti-apoptotic molecule. Though the integrated transcriptome showed that *Igf1* is highly expressed in neurons, another *Igf1* probe showed similar expression levels in neurons and microglia, and P90 SOD1^G93A^ microglia revealed 10.9-fold upregulation of *Igf1*; therefore, Igf1 is expressed in neurons and microglia in normal conditions, but the elevation of Igf1 in the spinal cords of symptomatic mutant SOD1 mice is presumably due to the activation of microglia. In a mouse ischemic injury model, activated/proliferating microglia have been reported to be the major source of Igf1 (Lalancette-Hebert et al., 2007). The receptor for Igf1, *Igf1r*, is relatively ubiquitously expressed and is not elevated in the spinal cords of mutant SOD1 mice. Administration of IGF1 with adeno-associated virus vectors can prevent motor neuron death and prolong lifespans in SOD1^G93A^ mice (Wang et al., 2018).

#### 3.6.3. Oligodendrocyte-enriched DEGs in SOD1-ALS mice

The two genes that are enriched in oligodendrocytes and included in the 207 DEGs were *Plat* and *Klk6* (Figure 3).

*Plat*-encoded tissue plasminogen activator (tPA) cleaves plasminogen to yield the active serine protease plasmin, which then proteolyzes its target proteins. While the plasmin proteolytic cascade is traditionally considered to function in fibrinolysis, the plasmin system may also be relevant to the clearance of Aβ, the causative protein of AD, a protein aggregation disease similar to ALS. Aggregated Aβ, in contrast to non-aggregated Aβ, increases the level of mRNAs encoding tPA. Purified plasmin degrades Aβ with physiologically relevant efficiency (Tucker et al., 2000).

*Klk6*-encoded kallikrein-related peptidase (Klk) 6 functions as a trypsin-like serine protease that can degrade various components of the extracellular matrix and is secreted mainly from oligodendrocytes. Klk6 is implicated in the turnover and uptake of extracellular alpha-synuclein (α-syn) species, the causative protein for Parkinson’s disease (Pampalakis et al., 2017). Furthermore, Klk6 can activate proteinase-activated receptor 1 (PAR-1) and proteinase-activated receptor (PAR)-2 in response to CNS damage, which leads to activation of several intracellular signaling pathways that may contribute to neurotoxicity (Mella et al., 2020). Expressions of the receptors for Klk6, *F2r* (PAR-1), and *F2rl1* (PAR-2) were not significantly altered in the spinal cords of mutant SOD1 mice. Upon *in vivo* administration of α-syn pre-formed fibrils, α-syn pathological accumulations are evident in the brains of both *Klk6*-knockout mice and wild-type mice without significant differences (Samantha Sykioti et al., 2021).

### 3.7. Microglia-enriched DEGs in SOD1-ALS mice

#### 3.7.1. Chemokines

Because of the large number of genes that were highly expressed in microglia in the 207 DEGs, we focused our analysis on several themes. First, based on the enrichment of proteins classified in the defense/immune protein and immunoglobulin receptor superfamily as shown in Figure 2A, we investigated chemokines, which are mainly involved in inflammatory cell migration.

The chemokine ligands *Ccl3, Ccl6, Ccl9*, and *Cxcl16*, and the chemokine receptor *Cx3cr1* were found in the 207 DEGs (Figure 4A). The integrated transcriptome indicated that all four chemokine ligands are predominantly expressed in microglia, so the elevation in the mouse spinal cord is considered to be primarily in microglia. However, for *Cxcl16*, there was an 85.7-fold increase in P120 SOD1^G93A^ astrocytes. In fact, it has been reported that *Cxcl16* expression is elevated in human glioblastoma multiforme and murine primary astrocytes (Ludwig et al., 2005), suggesting that astrocytes as well as microglia are involved in the upregulation of *Cxcl16* in the mutant SOD1 mouse spinal cords.

As for chemokine receptors, only *Cx3cr1* was included in the 207 DEGs, and its ligand *Cx3cl1* is also included for reference. The neuronal fractalkine (Cx3cl1) -microglial Cx3cr1 axis regulates microglial homeostatic functions and reduces microglial responses to inflammatory and injurious stimuli. In SOD1^G93A^ mice, deletion of *Cx3cr1* accelerates disease progression, indicating a protective role for CX3CR1 signaling in ALS (Cardona et al., 2006).

Chemokine systems vary considerably in the number and type of genes among species, reflecting rapid evolution. In Figure 4A, mouse Ccl6 and Ccl9 correspond to human CCL23 and CCL15, respectively.

#### 3.7.2. Microglial phenotypes

Next, we investigated genes associated with homeostatic microglia (HOM) and disease-associated microglia (DAM) as microglial phenotypes reported in neurodegenerative diseases (Figure 4B). The expression of representative DAM genes was increased. However, the expression of representative HOM genes was estimated to be somewhat decreased when microgliosis was taken into account, although it was elevated by 1.2- to 3.2-fold in all spinal cord samples. This trend was confirmed by P90 SOD1^G93A^ microglial data, consistent with our recent report (Sobue et al., 2021).

#### 3.7.3. Mac2, a microglial activation marker, is ectopically expressed in astrocytes during disease progression in SOD1-ALS mice

Analysis of P90 SOD1^G93A^ microglia and astrocytes suggested that *Lgals3* encoding galectin-3 (Mac2), a marker of microglial activation, was also ectopically transcribed in astrocytes in the mutant SOD1 mice as the disease progressed (Figure 4C). This 290.7-fold upregulation in astrocytes might have been due to contamination of nearby microglia by LCM, but the degree of upregulation was more pronounced in SOD1^G93A^ astrocytes compared to other representative markers of microglia, *Cd68, Aif1*, and *Itgam*, which ranged from 0.6- to 8.5-fold, suggesting an ectopic increase in expression. This could only be predicted by comparing the transcriptomes of SOD1^G93A^ cells (microglia and astrocytes) with the integrated transcriptome.

There are two possible causes of ectopic expression of Mac2 in astrocytes. One is as a result of the normal function of “non-professional phagocytes,” such as astrocytes (Nguyen et al., 2011) and Schwann cells (Reichert et al., 1994), which reactively phagocytose (Rotshenker, 2022). We have shown that in SOD1^G93A^ mice, astrocytes expressing Mac2 show accumulation of p62, ubiquitin, and mutant SOD1. This means that the upregulation of Mac2 is due to the disruption of the astrocyte lysosomal system, and an innate immune adaptor, TIR domain-containing adaptor inducing interferon-β (TRIF) may function to remove the disrupted astrocytes (Komine et al., 2018).

Mac2 is associated with activated microglia, since homeostatic microglia do not express Mac2, and the expression of Mac2 is considered either supportive or detrimental depending on the context. Deletion of galectin-3 has been shown to exacerbate disease progression in ALS mice (Lerman et al., 2012), while in an AD mouse model, the deletion of Mac2 is protective (Boza-Serrano et al., 2019). There are two possible reasons for this: one is that the effect of Mac2 on microglial activation may differ between disease models, and the other is that since Mac2 also plays roles other than microglial activation, such as repairing the lysosomal system in neurons (Jia et al., 2020), the sum of its effects on neurodegeneration may be different.

It is interesting that galectin-3 has been reported to interact with TREM2 in an AD mouse model (Boza-Serrano et al., 2019), which is a key molecule discussed later, and to regulate microglial activation in neurodegenerative diseases (Garcia-Revilla et al., 2022). The present method is one promising way to search for unknown molecules that are ectopically expressed under certain conditions.

### 3.8. Comprehensive transcriptomic overview in specific protein families in SOD1-ALS mice

While we extracted the 207 DEGs using a strict criterion of more than twofold change in all three strains of mutant SOD1 mice, it is difficult to manually check the fold changes in the mouse spinal cord for all genes, their integrated transcriptomes, and the fold changes in SOD1^G93A^ astrocytes/microglia. Fortunately, gene symbols are often partially common to each family, so we can search Supplementary Table 9 for genes belonging to a protein family of interest in a comprehensive manner.

As a featured protein family, we investigated the caspases (Supplementary Table 7), a family of proteases that play a central role in a number of processes including cell death and inflammation, and heat shock proteins (Figure 3 and Supplementary Table 8), which have been associated with unfolded protein responses by mutant SOD1 toxicity (Parakh and Atkin, 2016; Wright, 2020).

#### 3.8.1. Caspases

Only *Casp12*, encoding an inflammatory caspase, was included in the 207 DEGs. *Il1b*, encoding IL-1β, a major proinflammatory cytokine acting downstream of the inflammatory caspase, is added in the list for reference (Supplementary Table 7). Caspase 12 has been reported in association with ER stress (Wootz et al., 2004) and the apoptosis pathway (Wootz et al., 2006) in SOD1^G93A^ mice. In addition, caspase 12-knockout mice become resistant to ER stress-induced apoptosis (Nakagawa et al., 2000) and show enhanced bacterial clearance that increases resistance to sepsis (Saleh et al., 2006); therefore, caspase 12 is classified as an inflammatory caspase along with caspase 1 and 11, encoded by *Casp4*, in mice.

*Casp4* was not included in the 207 DEGs due to the unfulfilled FLAG-call criteria, but was quite elevated in mutant SOD1 mouse spinal cords. The official name of *Casp4* is “caspase 4, apoptosis-related cysteine peptidase,” although this gene and its encoded protein have historically been called caspase 11. *Casp4* is expressed in a microglia-specific manner when referring to the integrated transcriptome, and knockout of caspase 11 encoded by *Casp4* in SOD1^G93A^ mice does not affect neurodegeneration, inflammatory responses, or survival time, despite suppression of IL-1β (Kang et al., 2003). In addition, the human ortholog of caspase 4 is involved in the processing of TDP-43 (TAR DNA Binding Protein 43), which accumulates in the affected lesions of human sporadic ALS (Li et al., 2015; Yin et al., 2019).

#### 3.8.2. Heat shock proteins

Among the heat shock proteins, only two small heat shock proteins, Hsp25 (Figure 3) and heat shock protein β-6 (HspB6) (Supplementary Table 8), were found in the 207 DEGs.

*Hsph1*-encoded Hsp105, a member of the Hsp110 family, which we have reported interaction with mutant SOD1 (Yamashita et al., 2007), was relatively enriched in motor neurons when referring to the integrated transcriptome. Overexpression of *Hspa4l*-encoded HspA4l (Apg1), another member of the Hsp110 family, has been reported to extend the survival of SOD1^G85R^ mice (Nagy et al., 2016).

### 3.9. Predicted pathomechanism among different cell types in SOD1-ALS mice related to TREM2, apolipoprotein E, and lipoproteins

In the CNS, cholesterol is synthesized mainly in astrocytes and transported to neurons and other cells. Cholesterol is surrounded by apolipoproteins such as apolipoprotein E (ApoE) and apolipoprotein J (ApoJ/clusterin), which are the most abundant apolipoproteins in the brain that are mostly synthesized by astrocytes (Wang and Eckel, 2014) and released into the intercellular space as lipoproteins by ABC transporters (Li et al., 2022). In addition, microglia take up lipoprotein-Aβ complexes, which are formed in AD, efficiently *via* TREM2 (Yeh et al., 2016). Similarly, in mutant SOD1 mice, microglia are thought to be responsible for the clearance of extracellular aggregated SOD1.

Therefore, we analyzed the CNS cell types expressing the relevant transporters, apolipoproteins, and their receptors, as well as the expression changes in SOD1^G93A^ microglia and astrocytes (Figure 5A).

#### 3.9.1. Astrocytic changes

*Apoe* was upregulated by sevenfold in P120 SOD1^G93A^ astrocytes, and it was most abundantly expressed in astrocytes compared to other cell-types when referring to the integrated transcriptome. Cholesterol efflux is dependent on specific membrane transporters, such as *Abca1*, which mediate the formation of cholesterol-rich ApoE particles. *Abca1* was abundant in astrocytes and upregulated in P120 SOD1*^G93A^* astrocytes. *Clu* encodes clusterin, also known as apolipoprotein J, another lipoparticle protein similar to apolipoprotein E. Upregulation of *Apoe, Clu*, and *Abca1* in astrocytes suggests that lipoproteins secreted by astrocytes may be increased.

#### 3.9.2. Microglial changes

*Apoe* in P90 SOD1^G93A^ microglia was upregulated by 2.2-fold, which suggests that ApoE secreted from microglia may be increased. ApoE not only constitutes lipoproteins, but also is a major constituent of amyloid plaques in AD and promotes their aggregation (Ma et al., 1994; Parhizkar et al., 2019). It has been shown that microglia secrete ApoE in very poorly lipidated particles as compared to astrocytes (Huynh et al., 2019). Therefore, ApoE secreted from microglia may be involved in the aggregation of extracellular mutant SOD1. ApoE also binds to apoptotic neuronal cells and exerts an opsonin-like effect when microglia phagocytose them (Atagi et al., 2015).

Extracellular aggregated SOD1, which may form complexes with lipoproteins or ApoE, aggregated SOD1 itself, or debris from degenerating neurons, is potentially taken up by microglia, primarily *via* TREM2 expressed on the surfaces of microglia [see *Discussion* in Yeh et al. (2016)]. In fact, *Trem2*, which was included in the 207 DEGs and selectively expressed in microglia, was upregulated by threefold in P90 SOD1^G93A^ microglia. Therefore, Trem2-mediated phagocytosis was presumed to be enhanced.

Further, ApoE, which is upregulated in microglia, contributes to the activation of microglia through interactions with TREM2. The TREM2-APOE pathway induces a microglial phenotypic switch from a homeostatic to neurodegenerative phenotype (Krasemann et al., 2017).

#### 3.9.3. Predicted schema

We address the possible pathomechanisms among multiple cell types involving neurons, astrocytes, and microglia in ALS-linked mutant SOD1 transgenic mice predicted from our analysis (Figure 5B).

In a physiological state, cholesterol is transported from astrocytes to motor neurons and microglia *via* lipoproteins.

In a diseased state, mutant SOD1 aggregates are released from diseased motor neurons and bind to lipoproteins in the intercellular space. Thereafter, there are three possible reactions: (1) Microglia phagocytose SOD1 aggregate-lipoprotein complexes *via* TREM2, (2) Microglia phagocytose SOD1 aggregates with or without ApoE *via* TREM2, (3) Microglia phagocytose ApoE-bound motor neurons *via* TREM2. Then, TREM2-mediated phagocytosis changes the phenotype from HOM to DAM in microglia *via* ApoE signaling. Excessive DAM activation may contribute to exacerbation of ALS pathology. Ideally, we need transcriptome data at some different time points for analysis, but available data were limited.

### 3.10. Consideration in relation to sporadic ALS

Sporadic ALS with the formation of TDP-43-positive inclusions is characterized by the deposition of proteins of abnormal conformation, as with SOD1-ALS (Luh and Bertolotti, 2020), though our reasoning applies to only SOD1-ALS since we analyzed SOD1-ALS mice in this study. Therefore, we present the latest findings related to our study.

Lipoproteins that may form complexes with extracellular aggregated SOD1 include cholesterol esters, triglycerides, and phospholipids, in addition to free cholesterol. Dodge et al. have reported that lipid cacostasis occurs in the spinal cords of human ALS patients and SOD1^G93A^ mice, possibly as a result of impaired intracellular or intercellular lipid trafficking, and may be directly involved in the pathogenesis of the disease (Dodge et al., 2020). Also, Xie et al. (2022) have recently shown that TREM2 binds to TDP-43 and is involved in the neuroprotective effects of microglia in TDP-43-related neurodegeneration.

## 4. Conclusion

The basis for analyzing gene expression in multiple cell types that constitute a given tissue is to isolate samples by cell type and analyze each of them. However, as explained in the *Introduction*, there are problems associated with those methods. In this study, we established and utilized an integrated cell-type-specific transcriptome database to analyze changes in gene expression in spinal cord tissues consisting of multiple cell types, the main locus of the disease, to help elucidate the pathogenesis of ALS in mouse models. Furthermore, when SOD1^G93A^ individual cell data were combined, it became possible to predict functions across multiple cell types, though the time point from which the SOD1^G93A^ cells were collected in the available data was limited, and this is a limitation of this study.

In conclusion, we propose the following as potential therapeutic targets for regulation in SOD1-ALS: (1) transcription factors that induce microglia into proinflammatory phenotypes, such as Irf8 or C/EBPs, (2) molecules involved in the TREM2-APOE pathway, especially TREM2, and (3) astrocyte function involved in the dysregulation of lipids. We also hope that further research based on the list of 207 DEGs and any other entities presented in this report (Supplementary Table 9 has a complete list of all entities used as microarray probes) will identify novel and effective therapeutic targets.

## Data availability statement

The raw data from microarray hybridizations presented in this study are available on the Gene Expression Omnibus Database (Edgar et al., 2002), and the GEO accession number is GSE220705 (https://www.ncbi.nlm.nih.gov/geo/query/acc.cgi?acc=GSE220705).

## Ethics statement

The animal study was reviewed and approved by the Animal Care and Use Committee and Recombinant DNA Experiment Committee of Nagoya University and RIKEN.

## Author contributions

HY and KY designed the study and wrote the manuscript. HY performed the experiments and analyzed the data with support from OK and NF-T under the supervision of KY. All authors approved the final manuscript.

## Footnotes

1
www.ncbi.nlm.nih.gov/geo

2
http://www.pantherdb.org

3
https://www.proteinatlas.org

## Supplementary material

Immunohistochemistry The lumbar spinal cord was fixed by perfusion with 4% paraformaldehyde in phosphate buffer, cryoprotected for 48 h in 30% sucrose in phosphate-buffered saline (PBS), and frozen. Twelve-micron cryosections were immunostained with combinations of antibodies against the following proteins: GFAP (1:500, Sigma, United States), Iba1 (1:500, Wako, Japan), OSMR (1:200, R&D, United States), CNN3 (1:50, Santa Cruz Biotechnology, United States), and Dectin-1 (Clec7A) (1:100 AbD Serotec, United Kingdom) overnight followed by Alexa Fluor 488- or 546-conjugated anti-rabbit, anti-rat, anti-mouse, or anti-rabbit IgG (Thermo Fisher Scientific, United States or Jackson ImmunoResearch Laboratories, United States). The tissue was pre-incubated with 5% normal goat serum/0.3% Triton X-100 in PBS for 1 h at room temperature prior to incubation with primary antibodies. Sections were mounted with ProLong Antifade reagent (Thermo Fisher Scientific, United States) or VECTASHIELD mounting medium (VECTOR Laboratories, United States) and analyzed using a confocal microscope (LSM5 Exciter and LSM700, Carl-Zeiss, Germany).

Supplementary Figure 1 Confirmation of several genes in Figure 1E by immunohistochemistry. Lumbar spinal cord sections of symptomatic mutant SOD1 mice (SOD1^G37R^ or SOD1^G85R^) and control mice were immunostained. **(A)** Astrogliosis was revealed by GFAP immunostaining (red) in symptomatic mutant SOD1 mice, and oncostatin M receptor (green) encoded by *Osmr* was mainly upregulated in GFAP-positive astrocytes. **(B)** Calponin-3 (green) encoded by *Cnn3* was mainly upregulated in GFAP-positive astrocytes (red) in symptomatic mutant SOD1 mice. **(C)** C-type lectin domain family 7 member A (green, alternative name; Dectin-1) encoded by *Clec7a* was mainly upregulated in Iba1-positive microglia (red) in symptomatic mutant SOD1 mice. Microgliosis was evident in the symptomatic stage.

Supplementary Figure 2 Comparison of two popular normalization algorithms. **(A)** MAS5 as global normalization and **(B)** RMA as quantile normalization methods for CNS cell types (astrocytes, microglia, motor neurons, neurons, and oligodendrocytes). The x-axis represents the expression levels of genes as a log_2_ scale, and the *y*-axis represents the number of genes in each expression level. The distribution forms in MAS5 are different among cell types, though those in RMA are identical. Note that the trend in expression levels of *Gapdh* (red arrowheads) and *Actb* (blue arrowheads) are similar even among different cell types.

Supplementary Table 1 Characteristics of the mice used in the experiment. We obtained data from GEO in comparison 3. GEO, Gene Expression Omnibus.

Supplementary Table 2 Characteristics of the samples used for establishing the integrated transcriptome and SOD1^G93A^ cell-type transcriptome and accession numbers in GEO. **(A)** Integrated transcriptome. *Negative selection* indicates the isolation method for neurons using residual samples after removal of oligodendrocyte lineage cells and microglia by immunopanning, astrocytes by FACS, and endothelial cells and pericytes by immunopanning from brain cell suspensions. This partially includes motor neurons. Reported cell purity was >99.9%. **(B)** SOD1^G93A^ microglial transcriptome (P90). **(C)** SOD1^G93A^ astroglial transcriptome (P90, P120). **(D)** SOD1^G93A^ motor-neuronal transcriptome (P56, P101, P111, and P121). SC, spinal cord; LCM, laser capture microdissection; FACS, fluorescence-activated cell sorting.

Supplementary Table 3 Validation of our integrated transcriptome by comparison with a previously published microglial transcriptome. We cite here several genes in Supplementary Table 2, which showed highly specific markers for microglia relative to both CNS cell types and peripheral myeloid immune cells, from Chiu et al. (2013). Microglial fold-enrichment vs. CNS cell-types in columns 7 and 8 show how many times more each gene is expressed in microglia compared to its expression in the second most abundant CNS cell type [spinal cord, neurons, and astroglia in Figure 2A of Chiu et al. (2013)]. Numerical values were rounded to the nearest integer.

Supplementary Table 4 The SOD1^G93A^ cell type (microglia/astrocyte/motor neuron) transcriptome with raw data before calculating fold changes for each gene. Motor neuronal marker *Mnx1*, neuronal marker *Snap25*, and oligodendrocytic marker *Mog* are elevated in astrocytes collected with LCM. Also, astrocytic marker *Gfap* and oligodendrocytic marker *Mog* are elevated in motor neurons collected with LCM. These are most likely the result of sample contamination during collection and are a factor that should be considered when interpreting the value of fold change (F.C.). LCM, laser capture microdissection.

Supplementary Table 5 Total list of the 207 DEGs classified by molecular function using the PANTHER classification system. Column B is the top category of PANTHER protein class, column C is its child category, columns D and E are also in the child categories of their above category, and column F is the most specific (bottom) category of the gene. Column G shows gene symbols. The second row in columns H, J, L, and N to AC represents isolation methods for samples. Columns H, J, and L show gene expression fold changes in spinal cords of SOD1^G93A^, SOD1^G85R^, and SOD1^G37R^ mutant mice, respectively. Columns I, K, and M show corresponding *p*-values of fold changes. Columns N to R show the integrated transcriptome. Values in enriched cell types are shown in bold font. Columns S to Y show the SOD1^G93A^ cell-type transcriptome. Columns Z, AA, AB, and AC show gene expression fold changes in spinal cords of SOD1^G93A^ mutant mice at 70, 98, 112, and 126 days old, respectively, and accession numbers in GEO are GSM462476 to GSM462487 for mutant mice and GSM462455 to GSM462466 for control mice (three biological replicates each). Columns AD, AE, and AF are probe set IDs in the Affymetrix array, Entrez Gene ID, and gene title, respectively. Rows for gene categories not included in the 207 DEG are collapsed.

Supplementary Table 6 *ACTB* or *GAPDH* expression level by single-cell RNA-seq of human CNS cells. Data are from the Human Protein Atlas (https://www.proteinatlas.org). Note the approximately threefold difference in *ACTB* or *GAPDH* expression levels between high- and low-expressing cell types, respectively. nTPM, normalized transcripts per million.

Supplementary Table 7 Gene expression analysis of the caspase family. *Casp12*, encoding an inflammatory caspase, was included in the 207 DEGs. *Il1b*, encoding IL-1β, a major proinflammatory cytokine acting downstream of the inflammatory caspase, is added in the list for reference.

Supplementary Table 8 Gene expression analysis of the heat shock protein family. Among heat shock proteins, only two small heat shock proteins, Hsp25 and heat shock protein β-6 (HspB6), were found in the 207 DEGs. Other heat shock protein genes are included for reference.

Supplementary Table 9 A complete list of all entities (approximately 45,000) used as microarray probes. Column A shows probe set IDs in the Affymetrix array. Columns C, D, and E show gene expression fold changes in spinal cords of SOD1^G93A^, SOD1^G85R^, and SOD1^G37R^ mutant mice, respectively. Columns F to J show the integrated transcriptome. Columns K to Q show the SOD1^G93A^ cell-type transcriptome. Columns R, S, T, and U show gene expression fold changes in spinal cords of SOD1^G93A^ mutant mice at 70, 98, 112, and 126 days old, respectively. Columns V, W, and X are gene symbol, Entrez Gene ID, and gene title, respectively. Rows 3 to 56 are control and marker genes. Below row 57, entities are sorted by number and alphabetically.
