# Corrigendum: Comprehensive expression analysis with cell-type-specific transcriptome in ALS-linked mutant SOD1 mice: Revisiting the active role of glial cells in disease

**DOI:** 10.3389/fncel.2023.1160444

**Published:** 2023-03-03

**Authors:** Hirofumi Yamashita, Okiru Komine, Noriko Fujimori-Tonou, Koji Yamanaka

**Affiliations:** ^1^Department of Neurology, Japanese Red Cross Wakayama Medical Center, Wakayama, Japan; ^2^Department of Neurology, Graduate School of Medicine, Kyoto University, Kyoto, Japan; ^3^Department of Neuroscience and Pathobiology, Research Institute of Environmental Medicine, Nagoya University, Nagoya, Japan; ^4^Support Unit for Bio-Material Analysis, RRD, RIKEN Center for Brain Science, Wako, Japan; ^5^Department of Neuroscience and Pathobiology, Nagoya University Graduate School of Medicine, Nagoya University, Nagoya, Japan; ^6^Institute for Glyco-Core Research (iGCORE), Nagoya University, Nagoya, Japan

**Keywords:** transcriptome, amyotrophic lateral sclerosis, microarray, neurodegeneration, astrocytes, microglia, superoxide dismutase 1 (SOD1), lipids/lipoproteins

In the published article, there was an error in Figure 2 as published. The fourth column heading in Figure 2B was falsely written as “Spinal cord (G93A).” The correct column heading is “Spinal cord (G37R).” The corrected Figure 2 appears below.

**Figure 2:**
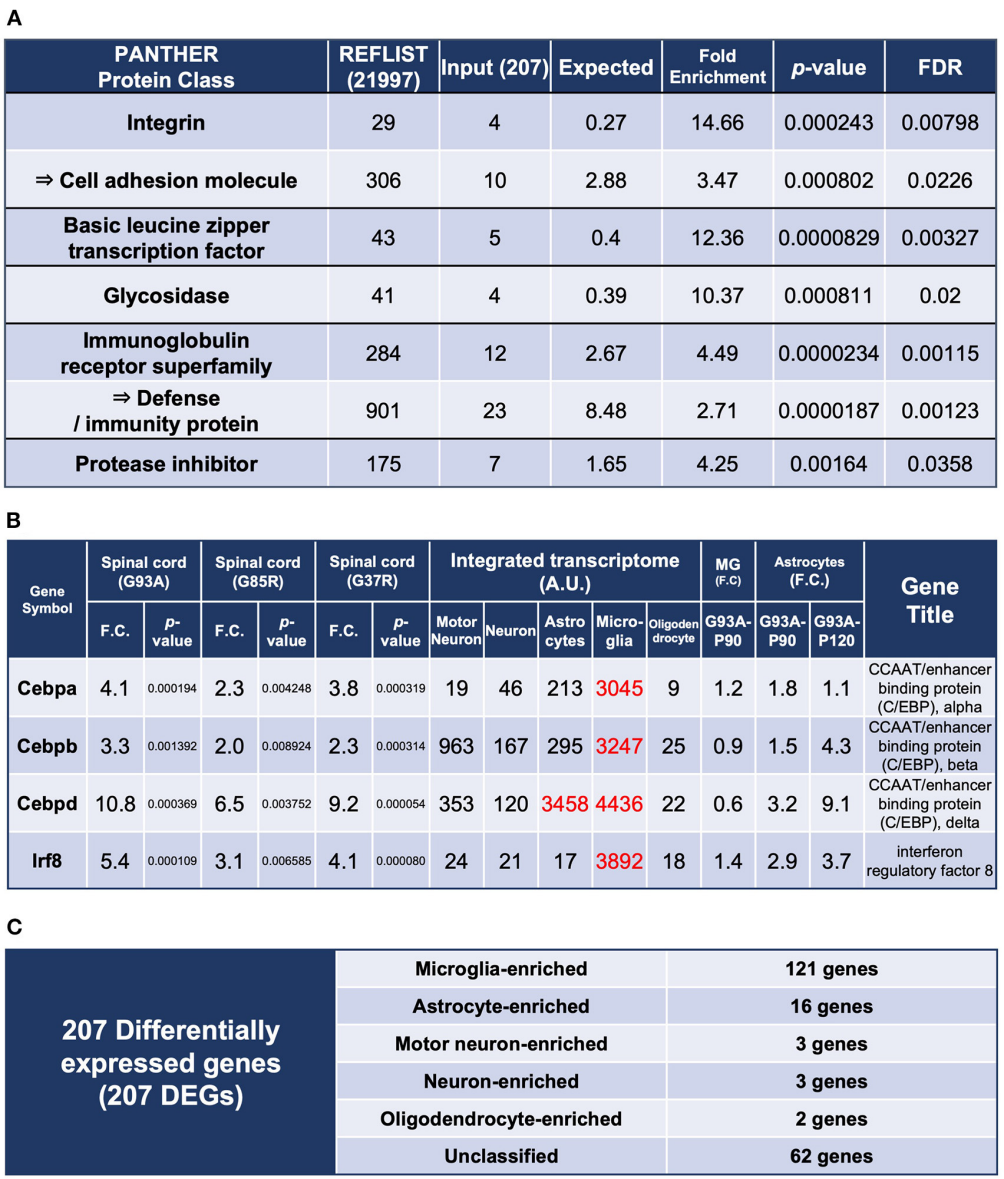
Enrichment analysis using the 207 DEGs and classification of each of the 207 DEGs by cell types with high expression. **(A)** Enrichment analysis using the 207 DEGs as an input. The first column shows the name of the PANTHER classification category. The second column shows the number of genes in the reference list that map to this particular PANTHER classification category. The total number of genes in the reference list is 21,997. The third column shows the observed number of genes in our input list that map to this PANTHER classification category. The fourth column shows the expected value, which is the number of genes we would expect in our list for this PANTHER category, based on the reference list. The fifth column shows the fold enrichment, which is the ratio of the value of column 3 (input: observed number) over that of column 4 (expected number). The sixth column shows the raw *p*-values. The seventh column shows the *q*-value (adjusted *p*-value, reflecting the false discovery rate) as calculated by the Benjamini-Hochberg procedure. *Cell adhesion molecule* is in the parent category of integrin, so it is shown in the row below. Similarly, *defense/immunity protein* is the parent category of *immunoglobulin receptor superfamily*. Therefore, they are indicated with arrows. **(B)** Representative transcription factors in the 207 DEGs. Three CCAAT/enhancer binding proteins and interferon regulatory factor 8 are shown. **(C)** All of the 207 DEGs were classified into cell types in which each gene is highly expressed; *Unclassified* are the genes that are not highly expressed in one particular cell type. MG, microglia.

In the published article, there was an error in Figure 3 as published. The *Tnfrs12a* values shown in Figure 3 are different from the data in 207 DEGs. The corrected Figure 3 appears below.

**Figure 3:**
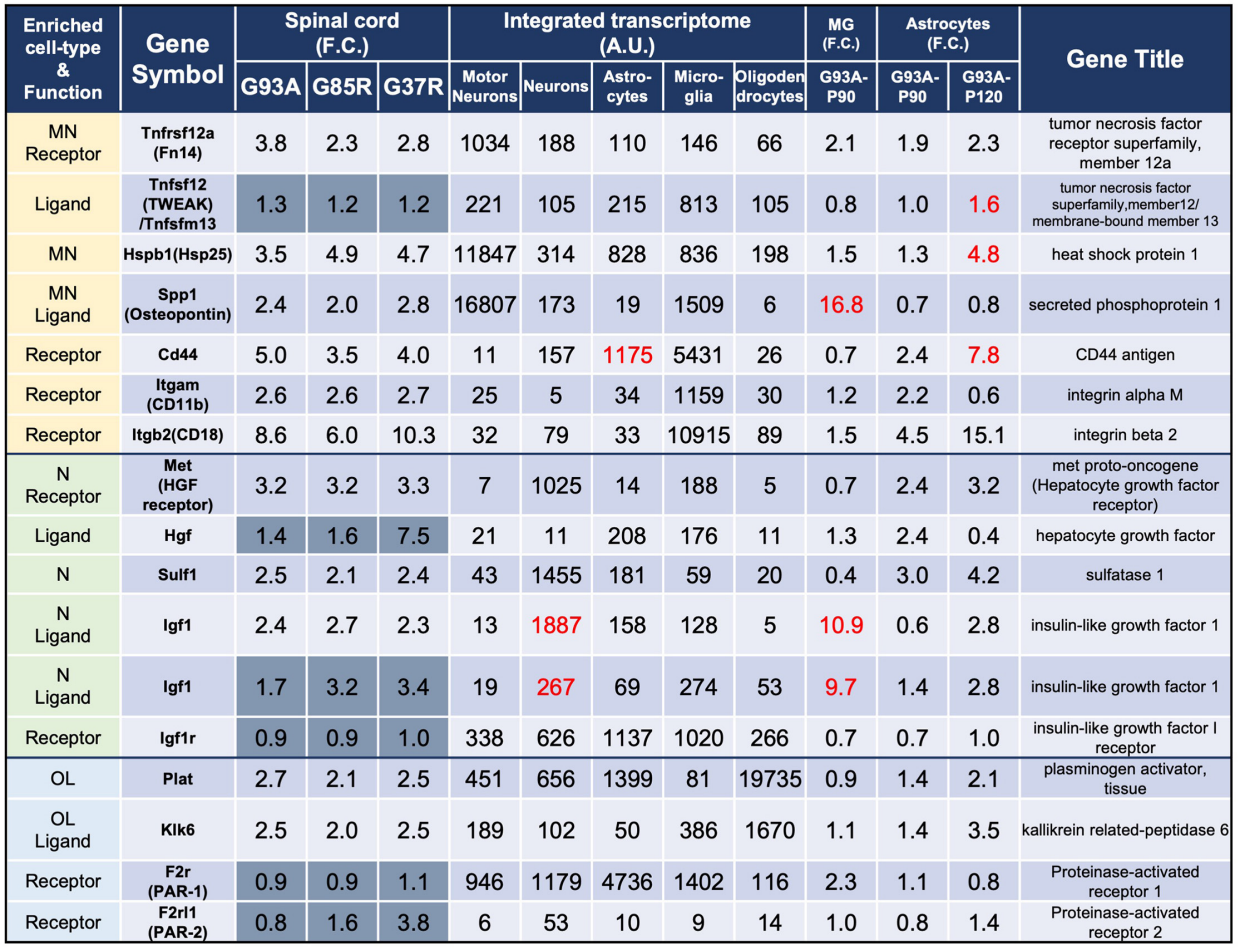
Representative motor neuron-enriched genes, neuron-enriched genes, and oligodendrocyte-enriched genes in 207 DEGs were analyzed using the integrated transcriptome and SOD1^G93A^ cell-type transcriptome. The 3rd to 5th columns show the fold changes in each gene expression in SOD1^G93A^, SOD1^G85R^, and SOD1^G37R^ mouse spinal cords compared to control samples, respectively. Genes with dark gray backgrounds in the 3rd to 5th columns that indicate fold changes (F.C.) are not included in the 207 DEGs. The 6th to 10th columns show the integrated transcriptome. The 11th column shows the fold change in expression of each gene in P90 SOD1^G93A^ microglia relative to control microglia. The 12th to 13th columns show the fold change in expression of each gene in SOD1^G93A^ astrocytes (P90 or P120) relative to control astrocytes. MN, motor neurons; N, neurons; MG, microglia; OL, oligodendrocytes.

In the published article, there was an error in Figure 5 and text as published. The fold change of *Abca1* in the spinal cords of SOD1^G85R^ mice shown in Figure 5 is 2.0, but this is a rounded value, which was 1.9779. *Abca1* is not included in Table S5, which shows the list of 207 DEGs with more than strict 2-fold change, although the 1.9779-fold change of SOD1^G85R^ mice is statistically significant as well as the 3.3-fold changes in the spinal cords of SOD1^G37R^ and SOD1^G93A^ mice. The corrected Figure 5 appears below.

**Figure 5:**
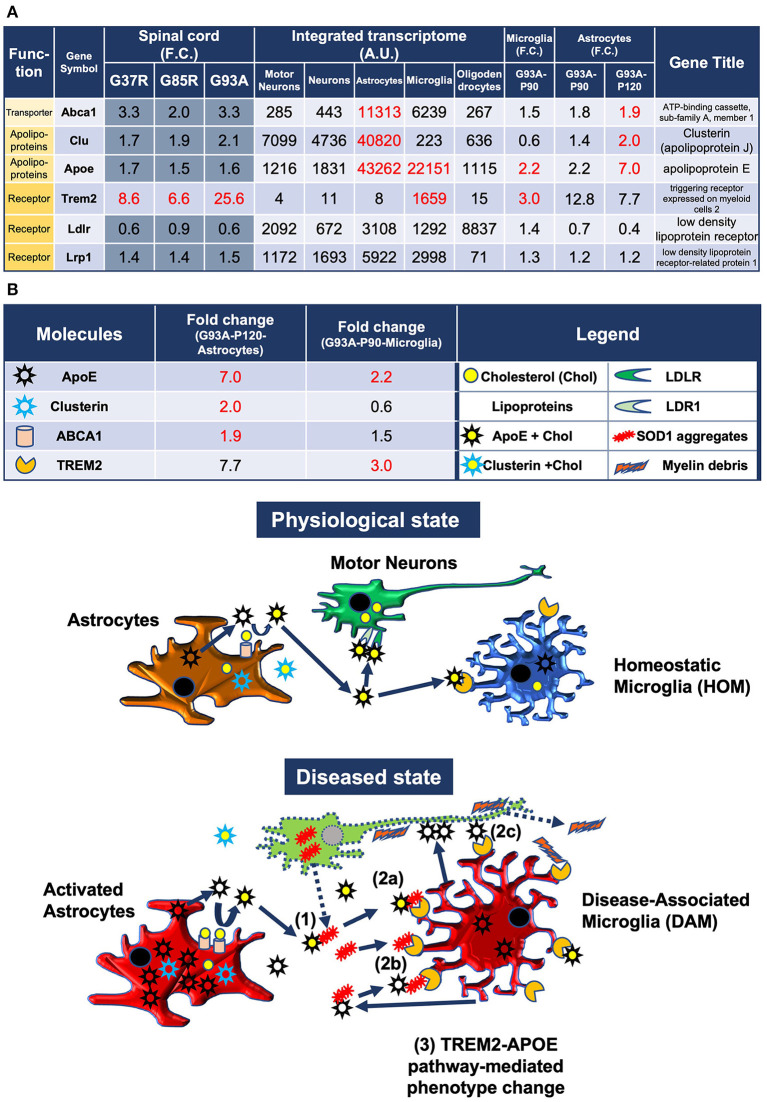
Model of the pathomechanism among different cell types in spinal cords of mutant SOD1 mice related to TREM2, apolipoprotein E, and lipoproteins. **(A)** Gene expression analysis of the relevant transporter *Abca1*, apolipoproteins *Apoe* and *Clu*, and receptors *Trem2, Ldlr*, and *Lrp1*, using integrated and SOD1^G93A^ cell type transcriptomes. **(B)** Physiological state: cholesterol is transported from astrocytes to motor neurons and microglia *via* lipoproteins. Diseased state: (1) Mutant SOD1 aggregates are released from diseased motor neurons and bind to lipoproteins in the intercellular space. (2a) Microglia phagocytose SOD1 aggregate-lipoprotein complexes *via* TREM2. (2b) Microglia phagocytose SOD1 aggregates with or without ApoE *via* TREM2. (2c) Microglia phagocytose ApoE-bound motor neurons *via* TREM2. (3) TREM2-mediated phagocytosis changes the phenotype from HOM to DAM in microglia *via* ApoE signaling. Excessive DAM activation may contribute to exacerbation of ALS pathology.

A correction has been made to Results and discussion, Predicted pathomechanism among different cell types in SOD1-ALS mice related to TREM2, apolipoprotein E, and lipoproteins, Astrocytic changes. This sentence previously stated:

“*Abca1* was found in the 207 DEGs and was abundant in astrocytes and upregulated in P120 SOD1^G93A^ astrocytes.”

The corrected sentence appears below:

“*Abca1* was abundant in astrocytes and upregulated in P120 SOD1^G93A^ astrocytes.”

In the published article, there was an error in Supplementary Table 5. Fold changes and *p*-value of three SOD1 mutant mice in Aspg, Casp12, Psmb8, Ctsd, Ctsc, Ctsh, Ctsl, Ctss, and Ctsz (214^th^ to 222^nd^ rows in uncollapsed sheet, columns H to M) were shifted one line by a handling error. The Supplementary Table 5 has been updated.

The authors apologize for this error and state that this does not change the scientific conclusions of the article in any way. The original article has been updated.

