# Supplementary material for: Comprehensive expression analysis with cell-type-specific transcriptome in ALS-linked mutant SOD1 mice: Revisiting the active role of glial cells in disease
Source: Front Cell Neurosci. 2023 Jan 4;16:1045647. doi: 10.3389/fncel.2022.1045647 (PMC9846815; doi:10.3389/fncel.2022.1045647)
Supplement: Immunohistochemistry — The lumbar spinal cord was fixed by perfusion with 4% paraformaldehyde in phosphate buffer, cryoprotected for 48 h in 30% sucrose in phosphate-buffered saline (PBS), and frozen. Twelve-micron cryosections were immunostained with combinations of antibodies against the following proteins: GFAP (1:500, Sigma, United States), Iba1 (1:500, Wako, Japan), OSMR (1:200, R&D, United States), CNN3 (1:50, Santa Cruz Biotechnology, United States), and Dectin-1 (Clec7A) (1:100 AbD Serotec, United Kingdom) overnight followed by Alexa Fluor 488- or 546-conjugated anti-rabbit, anti-rat, anti-mouse, or anti-rabbit IgG (Thermo Fisher Scientific, United States or Jackson ImmunoResearch Laboratories, United States). The tissue was pre-incubated with 5% normal goat serum/0.3% Triton X-100 in PBS for 1 h at room temperature prior to incubation with primary antibodies. Sections were mounted with ProLong Antifade reagent (Thermo Fisher Scientific, United States) or VECTASHIELD mounting medium (VECTOR Laboratories, United States) and analyzed using a confocal microscope (LSM5 Exciter and LSM700, Carl-Zeiss, Germany). [file Data_Sheet_1.pdf]

## Figure S1

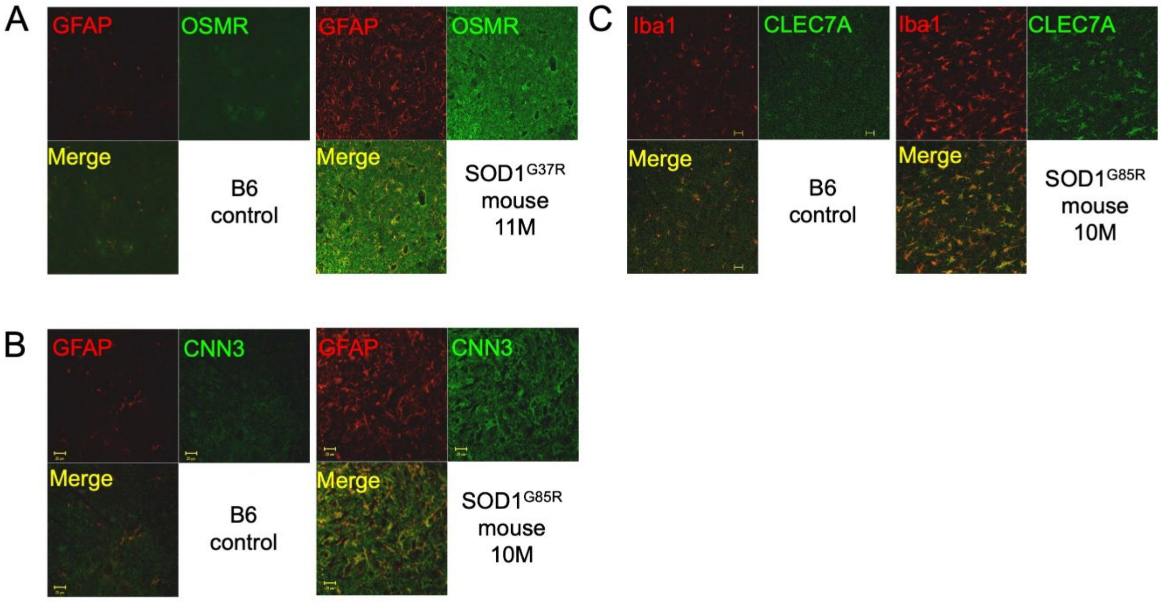


## Figure S2

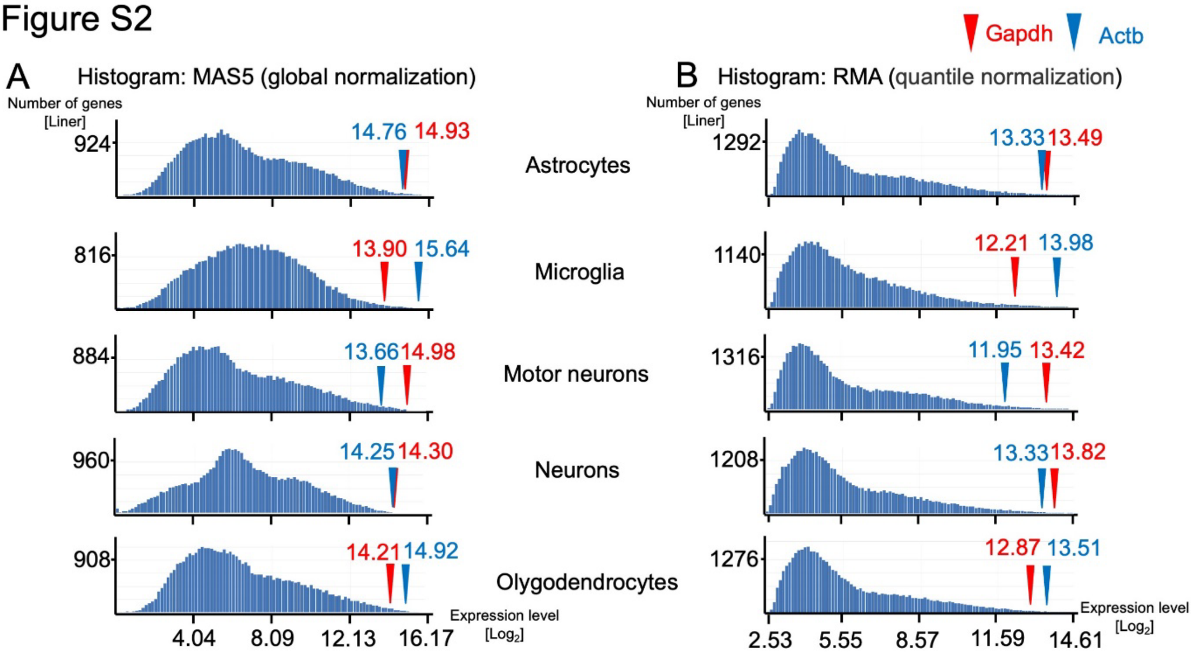

# Table S1

| | ALS mouse model (SOD1 activity), Age, Replicates | Control (SOD1 activity), Age, Replicates | Data source |
|---|---|---|---|
| Comparison 1 | G37R SOD1 Tg (increased), 11 months, N=3 | WT SOD1 Tg (increased), 11 months, N=3 | our experiment |
| Comparison 2 | G85R SOD1 Tg (unchanged), 11 months, N=3 | Non-Tg littermate (unchanged), 11 months, N=3 | our experiment |
| Comparison 3 | G93A SOD1 Tg (increased), 4 months, N=3 | Non-Tg littermate (unchanged), 4 months, N=3 | public deposit |
| | **GEO accession number** | | |
| | GSM462482, 462483, 462484 | GSM462461, 462462, 462463 | |

# Table S2

A

| Cell type | Sample source | Isolation methods | GEO accession number |
|---|---|---|---|
| Motor neurons | P60 ventral SC | LCM | GSM113702, 113703, 113704 |
| Neurons | P16 brain | Negative selection | GSM241897 |
| Astrocytes | P17 brain | S100β-FACS | GSM241912, 241914, 241926 |
| Microglia | P90 lumbar SC | CD11b-FACS | GSM2527942, 2527943, 2527944 |
| Oligodendrocytes | P16 brain | MOG-immunopanned | GSM241890, 241893, 241894 |

B

| Disease stages | Cell type | Sample source | Isolation methods | GEO accession number |
|---|---|---|---|---|
| Control | P90-Microglia (littermate) | P90 lumbar SC | CD11b-FACS | GSM2527942, 2527943, 2527944 |
| Early-symptomatic | P90-Microglia (G93A) | P90 lumbar SC | CD11b-FACS | GSM2527939, 2527940, 2527941 |

C

| Disease stages | Cell type | Sample source | Isolation methods | GEO accession number |
|---|---|---|---|---|
| Control | P90-Astrocytes (littermate) | P90 lumbar SC | LCM | GSM1694233, 1694234, 1694235 |
| Early-symptomatic | P90-Astrocytes (G93A) | P90 lumbar SC | LCM | GSM1694230, 1694231, 1694232 |
| Control | P120-Astrocytes (littermate) | P120 lumbar SC | LCM | GSM1694239, 1694240, 1694241 |
| Late-symptomatic | P120-Astrocytes (G93A) | P120 lumbar SC | LCM | GSM1694236, 1694237, 1694238 |

D

| Disease stages | Cell type | Sample source | Isolation methods | GEO accession number |
|---|---|---|---|---|
| Control | P56-Motor neurons (littermate) | P56 lumbar SC | LCM | GSM1128299, 1128300, 1128301, 1128302 |
| Pre-symptomatic | P56-Motor neurons (G93A) | P56 lumbar SC | LCM | GSM1128295, 1128296, 1128297, 1128298 |
| Control | P101-Motor neurons (littermate) | P101 lumbar SC | LCM | GSM1128315, 1128316, 1128317, 1128318 |
| Early-symptomatic | P101-Motor neurons (G93A) | P101 lumbar SC | LCM | GSM1128311, 1128312, 1128313, 1128314 |
| Control | P111-Motor neurons (littermate) | P111 lumbar SC | LCM | GSM1128331, 1128332, 1128333, 1128334 |
| Symptomatic | P111-Motor neurons (G93A) | P111 lumbar SC | LCM | GSM1128327, 1128328, 1128329, 1128330 |
| Control | P121-Motor neurons (littermate) | P121 lumbar SC | LCM | GSM1128347, 1128348, 1128349, 1128350 |
| Late-symptomatic | P121-Motor neurons (G93A) | P121 lumbar SC | LCM | GSM1128343, 1128344, 1128345, 1128346 |

# Table S3

| Gene Symbol | Integrated transcriptome (A.U.) | | | | | Microglia fold-enrichment vs. CNS cell-types (Microarray) Our data | Microglia fold-enrichment vs. CNS cell-types (RNAseq) From Chiu et al. | Gene Title |
|---|---|---|---|---|---|---|---|---|
| | Motor Neurons | Neurons | Astrocytes | Microglia | Oligoden-drocytes | | | |
| **Olfml3** | **25** | **36** | **59** | **12371** | **190** | **65** | **7** | **olfactomedin-like 3** |
| **Tmem119** | **89** | **133** | **40** | **16388** | **226** | **72** | **10** | **transmembrane protein 119** |
| **Siglec H** | **28** | **59** | **15** | **5212** | **88** | **59** | **9** | **sialic acid binding Ig-like lectin H** |
| Slc2a5 | 66 | 88 | 65 | 589 | 84 | 7 | 21 | solute carrier family 2 (facilitated glucose transporter), member 5 |
| Gal3st4 | 72 | 10 | 1172 | 6242 | 105 | 5 | 6 | galactose-3-O-sulfotransferase 4 |
| Csmd3 | 49 | 64 | 18 | 134 | 31 | 2 | 8 | CUB and Sushi multiple domains 3 |
| Slco2b1 | 83 | 58 | 80 | 20236 | 102 | 198 | 15 | solute carrier organic anion transporter family, member 2b1 |
| Gpr84 | 22 | 130 | 10 | 3107 | 86 | 24 | 15 | G protein-coupled receptor 84 |
| Lag3 | 9 | 18 | 26 | 901 | 31 | 29 | 15 | lymphocyte-activation gene 3 |
| Adora3 | 5 | 52 | 50 | 520 | 15 | 10 | 9 | adenosine A3 receptor///transmembrane and immunoglobulin domain containing 3 |
| Ccl4 | 64 | 235 | 127 | 3394 | 275 | 12 | 7 | chemokine (C-C motif) ligand 4 |
| Golm1 | 265 | 343 | 1180 | 9027 | 263 | 8 | 7 | golgi membrane protein 1 |
| P2ry13 | 67 | 49 | 55 | 12702 | 151 | 84 | 34 | purinergic receptor P2Y, G-protein coupled 13 |

# Table S4

| Cell type | Gene symbol | Integrated transcriptome (A.U.) | | | | | $SOD1^{G93A}$ cell-type transcriptome (A.U.) | | | | | | | | | | | | | | | | | | | | |
|---|---|---|---|---|---|---|---|---|---|---|---|---|---|---|---|---|---|---|---|---|---|---|---|---|---|---|---|
| | | | | | | | Microglia (FACS) | | | Astrocytes (LCM) | | | | | | Motor neurons (LCM) | | | | | | | | | | | |
| | | Motor Neuron | Neuron | Astrocyte | Microglia | Oligoden drocyte | G93A-P90-MG | NTg-P90-MG | G93A-P90-MG (F.C.) | G93A-P90-AS | NTg-P90-AS | G93A-P90-AS (F.C.) | G93A-P120-AS | NTg-P120-AS | G93A-P120-AS (F.C.) | G93A-P56-MN | NTg-P56-MN | G93A-P56-MN (F.C.) | G93A-P101-MN | NTg-P101-MN | G93A-P101-MN (F.C.) | G93A-P111-MN | NTg-P111-MN | G93A-P111-MN (F.C.) | G93A-P121-MN | NTg-P121-MN | G93A-P121-MN (F.C.) |
| General | **Actb** | 12978 | 19436 | 27715 | 51118 | 31061 | 52574 | 51118 | 1.0 | 41280 | 43363 | 1.0 | 39159 | 23950 | 1.6 | 23114 | 33182 | 0.7 | 26827 | 23536 | 1.1 | 22377 | 21593 | 1.0 | 44037 | 23428 | 1.9 |
| General | **Gapdh** | 32304 | 20116 | 31176 | 15320 | 18997 | 22191 | 15320 | 1.4 | 25016 | 25224 | 1.0 | 18770 | 20772 | 0.9 | 32096 | 49938 | 0.6 | 20432 | 23614 | 0.9 | 22629 | 21209 | 1.1 | 27912 | 22530 | 1.2 |
| Motor neuron | **Mnx1** | 891 | 25 | 45 | 14 | 6 | 4 | 14 | 0.3 | **148** | **200** | 0.7 | **86** | **122** | 0.7 | 392 | 317 | 1.2 | 243 | 488 | 0.5 | 147 | 367 | 0.4 | 116 | 279 | 0.4 |
| Neuron | **Snap25** | 21555 | 21916 | 344 | 124 | 149 | 38 | 124 | 0.3 | **13539** | **17024** | 0.8 | **7441** | **18893** | 0.4 | 42185 | 42198 | 1.0 | 33624 | 37514 | 0.9 | 34875 | 34188 | 1.0 | 16637 | 51644 | 0.3 |
| Astrocytes | **Gfap** | 136 | 69 | 3710 | 83 | 111 | 132 | 83 | 1.6 | 5269 | 1930 | 2.7 | 2542 | 273 | 9.3 | 423 | 825 | 0.5 | **2928** | **778** | 3.8 | **3730** | 479 | **7.8** | **6578** | 904 | **7.3** |
| Microglia | **Itgam** | 25 | 5 | 34 | 1159 | 30 | 1394 | 1159 | 1.2 | 33 | 15 | 2.2 | 26 | 47 | 0.6 | 7 | 25 | 0.3 | 32 | 15 | 2.1 | 22 | 18 | 1.2 | 42 | 28 | 1.5 |
| Oligodendrocyte | **Mog** | 598 | 6 | 14 | 152 | 25990 | 118 | 152 | 0.8 | **2621** | **2543** | 1.0 | **1740** | **1466** | 1.2 | **1874** | **2513** | 0.7 | **3192** | **3497** | 0.9 | **2761** | **2581** | 1.1 | **3411** | **2054** | 1.7 |
| Endocelial cells | **Ocln** | 29 | 86 | 78 | 78 | 14 | 86 | 78 | 1.1 | 74 | 63 | 1.2 | 94 | 113 | 0.8 | 217 | 225 | 1.0 | 142 | 208 | 0.7 | 162 | 192 | 0.8 | 180 | 178 | 1.0 |

# Table S6

| Cell type | Gene symbol | Excitatory neurons | Inhibitory neurons | Astrocytes | Microglia | Oligodendro-cytes |
|---|---|---|---|---|---|---|
| | | Single cell RNAseq (nTPM) | | | | |
| General | **ACTB** | 129.3 | 101.3 | 142.4 | 259.5 | 302.2 |
| General | **GAPDH** | 112.1 | 145.8 | 228.2 | 86.2 | 196.2 |

# Table S7

| Caspase Classes | Gene Symbol | Spinal cord (F.C.) | | | Integrated transcriptome (A.U.) | | | | | MG (F.C.) | Astrocytes (F.C.) | | Motor neurons (F.C.) | | | | Gene Title |
|---|---|---|---|---|---|---|---|---|---|---|---|---|---|---|---|---|---|
| | | G93A | G85R | G37R | Motor Neurons | Neurons | Astrocytes | Microglia | Oligodendrocytes | G93A-P90-MG | G93A-P90 | G93A-P120 | G93A-P56- | G93A-P101 | G93A-P111 | G93A-P121 | |
| Inflammation | **Casp1** | 2.0 | 1.6 | 2.0 | 48 | 54 | 26 | **1785** | 25 | 0.9 | 1.5 | 2.7 | 6.5 | 3.0 | 1.1 | 2.4 | caspase 1 |
| | **Casp4 (Casp11)** | **6.9** | **12.6** | **7.6** | 7 | 6 | 6 | **1328** | 6 | 1.5 | 4.3 | 16.9 | 2.0 | 1.6 | 15.2 | 14.8 | caspase4, apoptosis-related cysteine peptidase |
| | **Casp12** | **2.9** | **3.3** | **3.4** | 110 | 78 | 22 | 57 | 46 | 1.1 | 2.2 | 3.1 | 1.6 | 2.3 | 2.5 | 3.6 | caspase 12 |
| | **Il1b** | 0.9 | 1.6 | 2.2 | 50 | 71 | 66 | **6254** | 56 | 1.2 | 0.9 | 2.3 | 1.1 | 1.5 | 1.4 | 1.0 | interleukin 1 beta |
| Executioner of Apoptosis | **Casp3** | 1.6 | 1.3 | 1.2 | 261 | 1002 | 952 | 3157 | 1505 | 0.8 | 1.3 | 1.6 | 0.8 | 1.8 | 1.9 | 2.3 | caspase 3 |
| | **Casp6** | 2.2 | 1.1 | 1.3 | 63 | 121 | 811 | 420 | 275 | 1.3 | 1.1 | 1.5 | 3.4 | 1.4 | 1.6 | 5.8 | caspase 6 |
| | **Casp7** | 1.8 | 1.3 | 1.7 | 63 | 253 | 402 | 673 | 108 | 1.2 | 1.4 | 1.4 | 0.8 | 0.9 | 1.6 | 3.3 | caspase 7 |
| Initiator of Apoptosis | **Casp2** | 1.2 | 1.1 | 1.1 | 119 | 605 | 843 | 992 | 959 | 1.2 | 1.2 | 1.3 | 0.9 | 1.4 | 1.6 | 0.8 | caspase 2 |
| | **Casp8** | 1.4 | 2.2 | 1.8 | 62 | 76 | 115 | 1764 | 107 | 1.3 | 1.8 | 2.3 | 1.7 | 1.5 | 1.7 | 2.9 | caspase 8 |
| | **Casp9** | 1.0 | 1.1 | 1.1 | 533 | 282 | 664 | 588 | 342 | 1.0 | 1.2 | 0.7 | 0.7 | 1.0 | 1.0 | 0.8 | caspase 9 |
| | **Casp14** | 0.9 | 0.9 | 0.9 | 203 | 265 | 212 | 543 | 167 | 0.9 | 1.3 | 1.6 | 0.8 | 1.6 | 1.4 | 1.8 | caspase 14 |

# Table S8

| Family | Gene Symbol | Synonyms (or protein names) | Spinal cord (F.C.) | | | Integrated transcriptome (A.U.) | | | | | MG (F.C.) | Astrocytes (F.C.) | | Motor neurons (F.C.) | | | | Gene Title |
|---|---|---|---|---|---|---|---|---|---|---|---|---|---|---|---|---|---|---|
| | | | G93A | G85R | G37R | Motor Neurons | Neurons | Astrocytes | Microglia | Oligodendrocytes | G93A-P90-MG | G93A-P90 | G93A-P120 | G93A-P56- | G93A-P101 | G93A-P111 | G93A-P121 | |
| HspA | Hspa1a | Hsp70-3 | 1.5 | 1.2 | 1.3 | 580 | 1169 | 2070 | 27954 | 2084 | 0.6 | 0.6 | 1.5 | 2.7 | 1.5 | 1.2 | 1.1 | heat shock protein 1A |
| | Hspa1b | Hsp70-1 | 1.7 | 2.0 | 2.5 | 119 | 660 | 3268 | 16398 | 3277 | 0.8 | 3.0 | 2.6 | 1.6 | 1.9 | 0.5 | 1.1 | heat shock protein 1B |
| | Hspa2 | Hsp70-2 | 2.3 | 1.4 | 1.7 | 147 | 1001 | 1728 | 444 | 2155 | 0.9 | 1.2 | 1.8 | 1.1 | 1.3 | 1.5 | 2.0 | heat shock protein 2 |
| | Hspa5 | Grp78/BiP | 1.2 | 1.1 | 1.3 | 16006 | 11443 | 28164 | 30777 | 15067 | 1.4 | 1.0 | 0.8 | 1.1 | 1.1 | 1.2 | 1.2 | heat shock protein 5 |
| | Hspa8 | Hsc70/73 | 1.1 | 1.0 | 1.0 | 8363 | 18279 | 3798 | 9785 | 8769 | 0.8 | 0.7 | 0.8 | 1.1 | 1.0 | 0.9 | 0.7 | heat shock protein 8 |
| | Hspa9 | Hsp74/Grp75 | 1.1 | 0.9 | 1.1 | 7522 | 5050 | 5432 | 2382 | 3811 | 1.3 | 0.9 | 0.8 | 1.1 | 0.9 | 0.9 | 1.1 | heat shock protein 9 |
| | Hspa12a | - | 1.0 | 0.8 | 0.9 | 135 | 112 | 17 | 66 | 20 | 0.8 | 1.2 | 0.5 | 0.9 | 0.7 | 1.1 | 0.3 | heat shock protein 12A |
| | Hspa12b | - | 0.6 | 1.1 | 0.4 | 18 | 9 | 5 | 26 | 5 | 0.7 | 1.2 | 0.4 | 1.0 | 1.7 | 0.4 | 1.7 | heat shock protein 12B |
| | Hspa13 | - | 1.2 | 1.0 | 1.0 | 1775 | 2434 | 1611 | 1377 | 1889 | 1.0 | 0.8 | 0.7 | 1.6 | 1.0 | 1.1 | 0.9 | heat shock protein 70 family, member 13 |
| | Hspa14 | Hsp70-4 | 1.1 | 1.1 | 1.0 | 1036 | 1032 | 1063 | 1230 | 1165 | 1.0 | 0.9 | 0.7 | 1.0 | 0.8 | 0.7 | 0.7 | heat shock protein 14 |
| HspB | **Hspb1** | **Hsp25/27** | **3.5** | **4.9** | **4.7** | **11847** | **314** | **828** | **836** | **198** | **1.5** | **1.3** | **4.8** | **1.6** | **1.0** | **1.2** | **4.4** | **heat shock protein 1** |
| | Hspb2 | HspB2 | 1.6 | 1.4 | 1.0 | 28 | 172 | 84 | 40 | 135 | 2.0 | 1.1 | 1.8 | 1.3 | 1.3 | 0.6 | 2.0 | heat shock protein 2 |
| | Hspb3 | HspB3 | 0.7 | 2.4 | 2.5 | 6 | 134 | 34 | 314 | 65 | 0.9 | 1.6 | 4.6 | 2.0 | 2.7 | 1.2 | 1.2 | heat shock protein 3 |
| | **Hspb6** | **HspB6** | **2.5** | **2.6** | **2.5** | **388** | **229** | **1459** | **155** | **354** | **1.2** | **1.6** | **2.9** | **1.1** | **1.1** | **1.6** | **3.5** | **heat shock protein, alpha-crystallin-related, B6** |
| | Hspb7 | Hsp25-2 | 0.8 | 0.6 | 0.7 | 11 | 163 | 56 | 38 | 39 | 1.4 | 2.1 | 2.2 | 1.0 | 0.9 | 0.9 | 0.9 | heat shock protein family, member 7 (cardiovascular) |
| | Hspb8 | Hsp22 | 1.4 | 1.4 | 1.4 | 11728 | 139 | 1116 | 95 | 18 | 1.0 | 0.8 | 1.7 | 1.6 | 0.8 | 0.9 | 1.2 | heat shock protein 8 |
| | Hspb9 | HspB9 | 0.4 | 1.2 | 0.7 | 16 | 2 | 9 | 8 | 3 | 0.9 | 0.3 | 0.3 | 0.5 | 1.0 | 1.3 | 2.5 | heat shock protein, alpha-crystallin-related, B9 |
| | Hspb11 | - | 1.1 | 1.1 | 1.2 | 1535 | 857 | 866 | 670 | 2582 | 0.9 | 0.8 | 1.7 | 1.1 | 1.0 | 1.0 | 1.4 | heat shock protein family B (small), member 11 |
| HspC | Hsp90aa1 | Hsp90α | 1.2 | 0.9 | 1.0 | 24590 | 10514 | 5699 | 14260 | 12523 | 0.8 | 0.6 | 0.6 | 1.2 | 0.7 | 0.6 | 0.4 | heat shock protein 90, alpha (cytosolic), class A member 1 |
| | Hsp90ab1 | Hsp90β | 1.0 | 1.1 | 1.0 | 12484 | 12353 | 5353 | 3516 | 9650 | 1.3 | 0.9 | 0.9 | 1.1 | 0.9 | 0.8 | 0.9 | heat shock protein 90 alpha (cytosolic), class B member 1 |
| | Hsp90b1 | Grp94 | 1.2 | 1.1 | 1.2 | 1352 | 8078 | 5374 | 15644 | 6596 | 1.0 | 0.8 | 0.9 | 1.2 | 1.1 | 1.0 | 0.9 | heat shock protein 90, beta (Grp94), member 1 |
| Hsp60 | Hspd1 | Hsp60 | 1.0 | 1.0 | 1.1 | 18430 | 6555 | 4095 | 3197 | 4248 | 1.6 | 1.1 | 0.7 | 1.3 | 0.9 | 0.8 | 0.5 | heat shock protein 1 (chaperonin) |
| Hsp10 | Hspe1 | Hsp10 | 1.0 | 1.1 | 1.0 | 695 | 466 | 343 | 546 | 384 | 1.2 | 1.3 | 1.1 | 0.7 | 0.8 | 0.7 | 0.7 | heat shock protein 1 (chaperonin 10) |
| Hsp110 | Hspa4 | Apg2/Hsp110 | 1.2 | 0.9 | 1.1 | 4395 | 1467 | 1980 | 1772 | 3646 | 1.5 | 1.0 | 0.9 | 1.4 | 0.9 | 0.9 | 1.0 | heat shock 70 kDa protein 4 (heat shock protein 4) |
| | Hspa4l | Apg1/Osp94 | 0.8 | 0.8 | 0.9 | 4861 | 2235 | 1689 | 242 | 1151 | 0.9 | 0.9 | 0.3 | 1.4 | 0.8 | 0.8 | 0.3 | heat shock 70 kDa protein 4L (heat shock protein 4 like) |
| | Hsph1 | Hsp105/Hsp110 | 0.9 | 0.9 | 1.1 | 16847 | 9861 | 2861 | 6426 | 6777 | 0.8 | 0.9 | 0.7 | 1.3 | 0.9 | 0.9 | 0.6 | heat shock 105kDa/110kDa protein 1 |
